# Transcription Factor *GmMYB29* Activates *GmPP2C-37like* Expression to Mediate Soybean Defense Against *Heterodera glycines* Race 3

**DOI:** 10.3390/plants14233612

**Published:** 2025-11-26

**Authors:** Shuo Qu, Shihao Hu, Gengchen Song, Miaoli Zhang, Yingpeng Han, Weili Teng, Yongguang Li, Hui Wang, Haiyan Li, Xue Zhao

**Affiliations:** 1 Key Laboratory of Soybean Biology in Chinese Ministry of Education (Key Laboratory of Soybean Biology and Breeding/Genetics of Chinese Agriculture Ministry), Northeast Agricultural University, Harbin 150030, China; b220301003@neau.edu.cn (S.Q.); s230302008@neau.edu.cn (S.H.); s230302024@neau.edu.cn (G.S.); s240301082@neau.edu.cn (M.Z.); hyp234286@neau.edu.cn (Y.H.); twlneau@163.com (W.T.); yongguangli@neau.edu.cn (Y.L.); 2 National Center for Soybean Improvement, State Key Laboratory of Crop Genetics & Germplasm Enhancement and Utilization, Nanjing Agricultural University, Nanjing 210095, China

**Keywords:** *GmMYB29*, *GmPP2C-37like*, gene regulation, SCN resistance, soybean cyst nematode

## Abstract

Soybean cyst nematode (SCN, *Heterodera glycines*) is one of the major pathogens of soybean worldwide. We utilized the CHIP-Seq (chromatin immunoprecipitation sequencing) and RNA-Seq (RNA sequencing) data from the transgenic *GmMYB29* strain (*Glycine Max* roots). We then performed enrichment analysis using KEGG and GO to identify potential candidate genes within the promoter-binding region. A targeted regulatory relationship between the *GmMYB29* and *GmPP2C-37like* genes was further identified using the dual-luciferase Assay (Luciferase, LUC) and yeast one-hybrid Assay (Y1H). Hairy roots with target gene overexpression and gene-edited hairy roots were generated, and their resistance to soybean cyst nematode (SCN) was evaluated. Meanwhile, the presence of reciprocal genes with *GmPP2C-37like* was determined by the yeast two-hybrid library screening method. The targeting relationship between *GmMYB29* and *GmPP2C-37like* genes was further validated through the Y1H assay and LUC assay. Based on phenotypic assessments of SCN, transgenic soybean roots overexpressing *GmPP2C-37like* exhibited significantly enhanced resistance to SCN 3 compared to wild-type. Further analysis revealed that *GmPP2C-37like* collaborates with other regulatory factors to modulate soybean resistance against SCN. Yeast two-hybrid library (Y2H) screening identified 18 interacting proteins. These findings not only illuminate the functional role of *GmPP2C-37like* but also provide a foundation for dissecting its molecular network. Moreover, the results offer promising candidate genes for enhancing SCN resistance and optimizing soybean resilience through targeted genetic strategies.

## 1. Introduction

Soybean (*Glycine max* (Linn.) Merr.) originated in China and has been cultivated for over 5000 years. As an essential oil and protein crop [1], over 60% of China’s domestic soybean supply is imported. By 2025, the soybean planting area in the People’s Republic of China is expected to reach 1.07 million hectares, with an output of 23 million tons [2]. Soybean productivity in China still lags far behind that of major producers such as Brazil, the United States, and Argentina. Soil-borne diseases, insect pests, and other hazards pose serious threats to soybean growth, with inestimable global yield losses caused by diseases annually. Therefore, the prevention and control of plant diseases and insect pests have become imperative. Soybeans are affected by diseases, pests, and other factors in actual production, which severely impact both the quality and yield of soybeans. Soybean cyst nematode (SCN, *Heterodera glycines Ichinohe*), first discovered in North America, has a wide distribution. Its eggs can survive in the soil for 8–10 years with strong pathogenicity, making it one of the major soil-borne diseases of soybean. According to the different pathogenicities, SCN is divided into several different physiological species. SCN 1, SCN 3, and SCN 4 are mainly distributed in the main soybean production areas in China, with wide distribution and high abundance, among which the pathogenicity of SCN 4 is the strongest [3].

An in-depth understanding of the pathogenic mechanisms of these races and the resistance adaptation of soybeans requires the prior clarification of the complete life cycle of the SCN. The life cycle of the SCN is divided into three periods: the egg (J_1_), larval (J_2_), and adult (J_3_) stages. The most serious damage to soybean roots occurred during the J_2_ period when SCN dissolved the cell walls during migration to form syncytia, which in turn obtained nutrients from the host soybean to continue growth [4], which led to severe damage to soybean yields. In 1988, Riggs and Schmitt first established four discriminating SCN hosts, Pickett, Peking, PI88788, and PI90763, as antigenic identifiers of SCN physiological subspecies, using which 16 physiological subspecies of SCN could be classified [5]. The recognized international antigens against SCN are PI88788 and Peaking (PI548402), but over-usage of the antigens may cause SCN to become resistant to the antigens. Given the critical role of antigenic determinants in mediating SCN resistance at the agronomic level, the identification and exploitation of novel candidates are therefore pivotal for translating this phenotypic trait into targeted genetic and biotechnological breeding strategies for SCN-resistant soybeans [6]. Traditional breeding is characterized by a long cycle, and favorable traits are tightly linked to undesirable ones, making it challenging to select soybean germplasm with multiple superior agronomic traits and SCN resistance. With the advancement of genetic engineering, the exploitation of SCN resistance genes via molecular biology techniques has emerged as an effective auxiliary method for conventional breeding [7]. Resistance to SCN is controlled by multiple genes that function together to regulate complex quantitative traits, and two of the more recognized resistance sites are the rhg1 and Rhg4 sites. Cook et al. showed that rhg1 locus resistance is determined by a tandem arrangement of three genes, including a transporter with the structural domain of a tryptophan/lysine permease, a SNAP protein, and a protein with the structural domain of a trauma-inducible protein [8]. It has been demonstrated that resistance at the Rhg4 locus is determined by serine hydroxymethyltransferase (*GmSHMT*), and that single-nucleotide polymorphism (SNP) mutations in the *GmSHMT*-encoding gene between resistant and susceptible soybean varieties lead to significant differences in the substrate-binding capacity of the *GmSHMT* enzyme, thereby affecting the catalytic activity of the mature protein. This affects folate homeostasis in the syncytium, resulting in changes to the internal environment of syncytium cells and ultimately leading to SCN death [9].

Beyond the well-characterized rhg1 and Rhg4 resistance loci, emerging studies have turned to other gene families to explore their potential roles in mediating SCN resistance, and *PP2C* family genes stand out due to their extensive distribution across plant species. *PP2C* family genes are widely present in plants. With the continuous improvement of plant whole-genome sequencing technology, *PP2Cs* were successfully identified from the sequencing results of approximately 8150 mosses and stone pines [10]; the number of *PP2C*-encoded genes is 80 in *Arabidopsis thaliana*, more than 90 in rice [11], 97 in maize [12], and 131 in oilseed rape [13]. It can be seen that the number of *PP2C* genes tends to increase during the evolution of plants from lower to higher levels, suggesting that this gene family satisfies the needs of plants during the evolutionary process [10]. The *PP2C* family is organized into 13 subfamilies (A–M) and unclassified members; subfamily A is the most extensively studied of the *PP2C* subfamilies. For instance, in *Arabidopsis thaliana*, ABI1 (ABA insensitive 1) and ABI2 were first found to play a negative regulatory role in abscisic acid (ABA)-related signal pathways involved in the regulation of stomatal closure, seed germination, and plant growth [14]; members of subfamily B are usually involved in mitogen-activated protein kinase (MAPK) pathway regulation. More and more other subfamily members have been reported to play important roles in plant response to stress and regulation of growth and development. Pathogenic bacteria, insect pests, and other biotic stresses may threaten plant growth. Plants formed a series of mechanisms to resist these stresses, in which *PP2C* also plays a role. Stomata are natural channels for the entry of some bacteria and fungi [15], and ABA-mediated stomatal closure inhibits the entry of pathogenic bacteria and is a positive regulator against biological stresses [16]. *PP2C-1* is involved in the MAPK signaling pathway as a negative regulator in plant response to biotic stresses, and overexpression of *PP2C-1* resulted in reduced MAPK activity, decreased ethylene levels, and suppression of plant immunity to Botrytis cinerea (gray mold fungus) [14]. In contrast, the ap2c1 mutant produces large amounts of jasmonic acid (JA) when damaged and shows higher resistance to insect pests such as leaf mites [17]. In rice, XB15 encodes a *PP2C* that negatively regulates the receptor kinase XA21-mediated innate immune response [18].

Our laboratory previously identified the *GmUGT88A1* gene via a multi-omics approach. Using yeast one-hybrid (Y1H) and dual-luciferase reporter assays, the interaction between *GmUGT88A1* and its cognate transcription factor *GmMYB29* was characterized [19]. Building on this foundation, we further identified the target genes of *GmPP2C-37like* via dual-luciferase reporter and yeast one-hybrid (Y1H) assays, based on chromatin immunoprecipitation sequencing (CHIP-Seq) and RNA sequencing (RNA-Seq) data derived from root tissues of *GmMYB29*-overexpressing soybean lines under SCN 3 stress. GUS staining was employed to assess the binding affinity between *GmMYB29* and *GmPP2C-37like*. The coding sequence (CDS) of *GmPP2C-37like* was cloned, and subsequent bioinformatics analyses were conducted for this gene. Overexpression and gene-editing vectors were successfully constructed, and transgenic soybean hairy roots were generated. Disease-resistance assays confirmed that *GmPP2C-37like* positively regulates soybean resistance to SCN 3. Subcellular localization analysis was carried out to determine the primary subcellular compartment where this gene exerts its functional activity. To explore potential protein–protein interactions of *GmPP2C-37like*, a yeast two-hybrid (Y2H) library screening assay was performed. The expression patterns of the identified interacting genes were also analyzed. The overall objective is to clarify the role of *GmPP2C-37like* in the mechanism of SCN resistance, thereby providing a theoretical reference for related research.

## 2. Results

### 2.1. CHIP-Seq Sequencing Analysis of the GmMYB29 Strain

**Figure 1:**
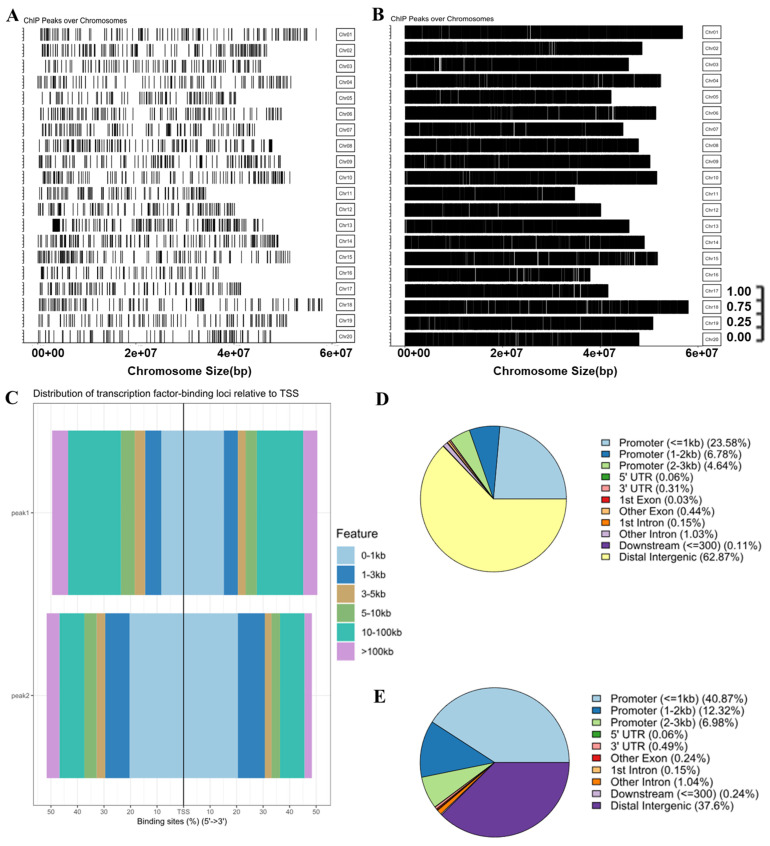
*GmMYB29* transcription factor binding positions. Coverage of 20 chromosomes. (**A**): Chromosome coverage map of wild-type (WT) soybean roots; (**B**): Chromosome coverage map of transformed OX-*GmMYB29* soybean roots; (**C**): Binding position histogram, peak_1_: OX-*GmMYB29* soybean root sequencing results; peak_2_: WT soybean root sequencing results; (**D**): OX-*GmMYB29* transcription factor binding target gene pie chart; (**E**): WT binding target gene position pie chart.

The results showed that the immunoprecipitation-bound DNA sequences in wild-type (WT) soybean roots exhibited low enrichment across all 20 chromosomes. In the absence of the GFP (green fluorescent protein)-targeting antibody, co-immunoprecipitation yielded an extremely low level of protein enrichment, which effectively excluded false positive results (Figure 1A). The genome-wide peak distribution across the 20 chromosomes of *GmMYB29*-transgenic soybean roots was comprehensive, with the target protein showing robust binding to all chromosomes (Figure 1B). Collectively, the test samples were of high quality, and the results are reliable for subsequent analyses to identify transcription factor-specific binding sites.

Peak 1 in the bar graph is the promoter-binding region of OE-*GmMYB29* root sequencing; about 30.36% of the OE-*GmMYB29* group fell in the promoter region, and the enrichment number of genes in the test group was 63,936. Peak 2 in the bar graph is the WT group; about 53.19% of the WT group fell in the promoter region, and the number of genes enriched in the WT was 3279 (Figure 1C, Table S15). WT group 0–1 kb promoter region accounted for 40.87%, promoter 1–2 kb accounted for 12.32%, promoter 2–3 kb accounted for 6.98%, 5′ UTR accounted for 0.06%, 3′ UTR accounted for 0.49%, and distal intergenic accounted for the largest proportion: 37.60%. Sequences falling in the 0–2 kb range accounted for 53.19% of the total (Figure 1D, Table S15). *OE-GmMYB29* group 0–1 kb promoter region accounted for 23.58%, promoter 1–2 kb accounted for 6.78%, promoter 2–3 kb accounted for 4.64%, 5′ UTR accounted for 0.06%, 3′ UTR accounted for 0.31%, and the upper end of the intergenic accounted for the largest proportion: 62.87%. Sequences falling in 0–2 kb accounted for 30.36% of the overall (Figure 1E, Table S15). The results indicated that the binding protein encoded by *GmMYB29* binds to the promoter region more extensively, and further enrichment analyses are needed to determine the regions where *GmMYB29* transcription factors bind to the promoter, which will lay important groundwork for the subsequent study of downstream target genes.

### 2.2. GO and KEGG Enrichment Analysis

**Figure 2:**
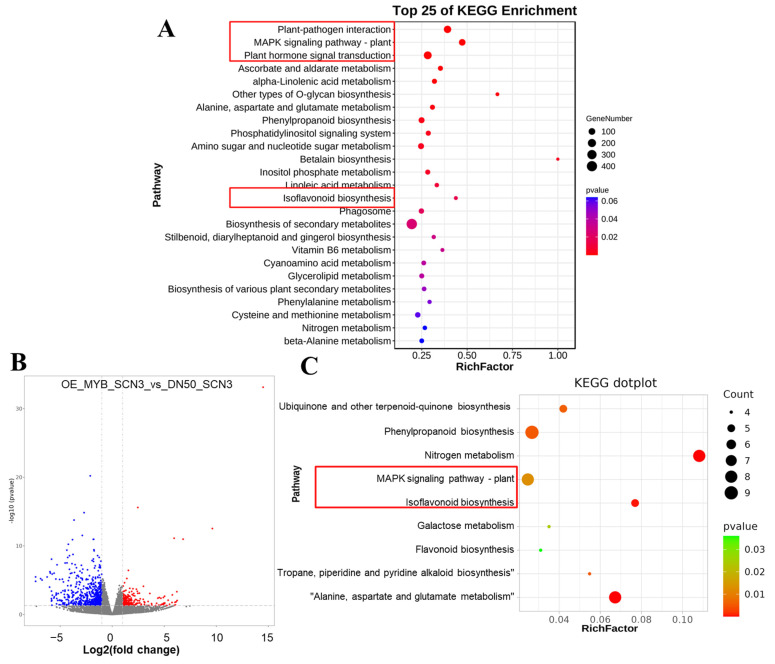
KEGG enrichment bubble plot. (**A**): *GmMYB29*-related genes are enriched in plant signal transduction synthesis pathway, MAPK signal transduction pathway, phytohormone signal transduction pathway, and isoflavone biosynthesis pathway; (**B**): Volcano plot of OX_MYB_SCN 3_vs_DN50_SCN 3 transcriptome; red dots, up-regulated genes; blue dots, down-regulated genes; gray dots: genes with no differential expression; (**C**): KEGG enrichment plot of shared genes screened by combined analysis of RNA-Seq and CHIP-Seq; The red box highlights the enriched pathways of differentially expressed genes (DEGs) of key interest.

Based on the specific binding site information within the 0–2 kb region upstream of the transcription start site (TSS) from CHIP-Seq data, 9972 candidate genes were subjected to KEGG and GO enrichment analyses. GO enrichment results were analyzed by pathway annotation under two categories (biological process and molecular function), with genes lacking GO annotations excluded. A total of 5964 enrichments were observed in biological process and 590 in molecular function (Figure S1A). Among these, genes encoding nucleus-localized functional proteins were enriched 159 times, while genes associated with actin filaments and the Piccolo NuA4 histone acetyltransferase complex accounted for 0.064% of the enriched genes (Figure S1B). GO functional annotations of candidate genes within 2000 bp of the TSS were classified into 42 functional subgroups, which were further grouped into three major categories: biological process, cellular component, and molecular function (Figure S1C). A total of 25 pathways were enriched, with a focus on 5 key pathways: plant hormone signal transduction (ko04075), MAPK signaling pathway–plant (ko04016), plant–pathogen interaction (ko04626), and isoflavonoid biosynthesis (ko00943). Potential candidate genes for further transcription factor-DNA interaction studies were identified through functional annotation and literature mining (Figure 2). In the KEGG enrichment results, 142 candidate genes were assigned to plant–pathogen interaction (ko04626), accounting for 9.59% of the total genes in this pathway. For MAPK signaling pathway–plant (ko04016), 95 candidate genes were identified, representing 6.42% of the pathway’s total genes. Plant hormone signal transduction (ko04075) included 175 candidate genes, accounting for 11.82% of the pathway’s total. Additionally, 7 candidate genes were annotated to isoflavonoid biosynthesis (ko00943), accounting for 0.74% of the total genes in this pathway (Figure S2, Table S11).

### 2.3. RNA-Seq & ChIP-Seq Integrative Analysis

RNA-Seq results showed that there was a total of 936 differentially expressed genes (DEGs) between *OX-GmMYB29* (with recipient material DN50) and wild-type (WT, DN50) under SCN 3 stress (After 14 d of stress treatment, the number of SCN-J2s stage reaches its peak, which causes the most significant damage to plant roots). Among these DEGs, 269 were up-regulated, while the number of down-regulated differentially expressed genes listed in the table below is 667. The volcano plot visualization of these DEGs is shown in Figure 2B.

The co-enrichment analysis of RNA-Seq and CHIP-Seq data indicated co-localization in the MAPK signaling pathway–plant and isoflavonoid biosynthesis pathways. Notably, the results showed that the promoter of LUC11 (*Glyma.18G035000* and *GmPP2C-37like*) is targeted and bound by the *GmMYB29* transcription factor; furthermore, RNA-Seq data revealed that this gene is up-regulated. This finding confirms that overexpression of *GmMYB29* can induce the up-regulation of *GmPP2C-37like*, which is involved in the MAPK signaling pathway and plant hormone signaling. (Figure 2C, Table S12)

### 2.4. Enrichment Analysis to Validate Candidate Genes

Twelve candidate genes in four metabolic pathways were proposed to be validated. The three candidate genes screened in the plant signal transduction synthesis pathway were *Glyma.01G222300* (K13425), *Glyma.18G087800* (K13457), and *Glyma.06G187200* (K18875), and three candidate genes screened in the MAPK signal transduction pathway were *Glyma.18G035000* (K14497), *Glyma.08G223400* (K13413), and *Glyma.15G051600* (K13416); the four candidate genes of phytohormone signaling pathway were *Glyma.15G062400* (K13449), *Glyma.02G125600* (K14487), *Glyma.05G081900* (K14498), and *Glyma.13G030300* (K00454); the two candidate genes in the isoflavone biosynthesis pathway were *Glyma.07G202300* (K13257) and *Glyma.18G267900* (K13262) (Table S8). Dual-luciferase complementation assay as well as yeast one-hybrid assay were performed to further determine whether there is a targeting relationship between the transcription factor *GmMYB29* and the promoter regions of the candidate target genes, and to further determine that multiple genes co-regulate SCN disease.

### 2.5. Luciferase Complementation Assay

**Figure 3:**
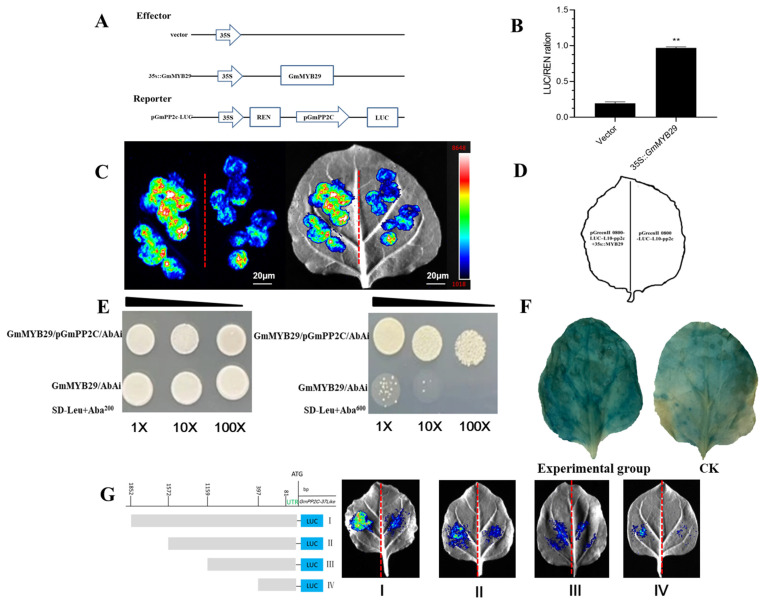
*GmMYB29* and target gene promoter interaction analysis. (**A**): LUC reporter vector *(proGmPP2C-37like*::LUC) and effector vector (*35sPro*::*GmMYB29*); (**B**): pCAMBIA3300-35S::*GmMYB29* and *GmPP2C-37like* bi-luciferase complementation assay; (**C**): *GmMYB29* activates the expression of *proGmPP2C-37like*::LUC in tobacco leaves; (**D**): Schematic diagram of tobacco injection. (**E**): Y_1_Hgold analysis showed that *GmMYB29* combined with *GmPP2C* promoter; yeast culture is continuously diluted at 1:10:100 on the plate. (**F**): GUS staining to explore the binding activity of *GmMYB29* to *proGmPP2C-37like* was analyzed. Experimental group: pBI121-*ProGmPP2C-37like* and pCAMBIA3300-*MYB29 Agrobacterium* GV3101 (psoup-19) were co-transformed into tobacco leaves; CK:pBI121-*ProGmPP2C-37like Agrobacterium* GV3101 (psoup-19) transformed into tobacco leaves. (**G**): Promoter truncation assay: The promoter region of the *GmPP2C-37like* gene was truncated into fragments I–IV with lengths of −397, −1159, −1572, and −1852 bp, respectively. The truncated DNAs were recombined into the LUC reporter vector (*proGmPP2C-37like*::LUCI–IV) and co-transformed into tobacco leaves together with the effector vector (35sPro::*GmMYB29*). ** significant at *p* < 0.01.

To further determine whether the *GmMYB29* gene interacts with the target gene, we validated it using a dual-luciferase reporter system. We found that LUC11 (*Glyma.18G035000* and *GmPP2C-37like*) interacted significantly with pCAMBIA3300-35S: *GmMYB29*. The combination of 35S::*GmMYB29* and LUC11 was able to significantly observe fluorescence and detected luciferase activity, while the negative control fluorescence intensity was weak. In statistics, if the interaction signal (fluorescence intensity) in the experimental group is twice or more than that in the control group, a “significant interaction” can be determined. In the present study, the fluorescence intensity in the experimental group was approximately 8000 μmol·m^−2^·s^−1^, with a wide coverage area, whereas that in the control group was approximately 1000 μmol·m^−2^·s^−1^. The fluorescence intensity of the experimental group was eight times that of the control group, and the results of *t*-test analysis showed highly significant differences. The results indicated that *GmMYB29* was able to bind to the LUC11 promoter and promote its transcription. The LUC/REN activity ratio showed 35S::*GmMYB29* transcriptional regulation was significantly higher than the empty vector, indicating a strong regulatory effect of *GmMYB29* (Figure 3A–D).

### 2.6. Promoter Truncation Analysis Identifies the GmMYB29-Binding Motif in the LUC11 Promoter

The results showed that the promoter truncations at −1852 bp and −1572 bp relative to the transcription start site (TSS) could effectively bind to the GmMYB29 protein. This binding resulted in significantly higher fluorescence intensity in the experimental groups than in the control group in tobacco leaves. In contrast, the truncations at −1159 bp and −397 bp lost their ability to bind GmMYB29, leading to a gradual decrease in fluorescence intensity in the experimental groups. These findings demonstrate that the critical binding region of GmMYB29 to the *LUC11* promoter is primarily located between −1159 bp and −1572 bp (Figure 3G).

### 2.7. Yeast One-Hybrid

We determined the critical growth concentration of Aureobasidin A (AbA) for the bait strains by inoculating them on SD/-Ura plates containing 200 ng/mL, 400 ng/mL, or 600 ng/mL AbA. The pAbAi strains grew normally on SD/-Ura plates; however, with increasing AbA concentration, the bait yeast strains failed to grow on SD/-Ura medium containing 600 ng/mL AbA. This indicates that the bait sequence is not recognized by endogenous yeast transcription factors, so a yeast one-hybrid screen was performed using 600 ng/mL AbA. The coding sequence (CDS) of *GmMYB29* was cloned and ligated into the expression vector pGADT7, and the resulting recombinant vector pGADT7-*GmMYB29* was co-transformed with the bait vector pAbAi-*ProGmPP2C-37like* into Y1Hgold yeast cells. The results showed that the co-transformed cells grew normally on the selective medium containing 600 ng/mL AbA, demonstrating that GmMYB29 interacts with the *GmPP2C-37like* promoter sequence (Figure 3E).

### 2.8. GUS Staining Assay Confirms Specific Binding of GmMYB29 to the GmPP2C-37like Promoter

GUS staining assay was performed to evaluate the binding activity of *GmMYB29* to the *GmPP2C-37like* promoter (*proGmPP2C-37like*). The results showed that the GUS staining intensity in the experimental group was deeper than that in the control group (CK), demonstrating that the protein encoded by *GmMYB29* can specifically bind to the *GmPP2C-37like* promoter region, confirming a targeted interaction (Figure 3F).

### 2.9. qRT-PCR Analysis Reveals Stress- and Hormone-Induced Expression of GmPP2C-37like in Soybean

**Figure 4:**
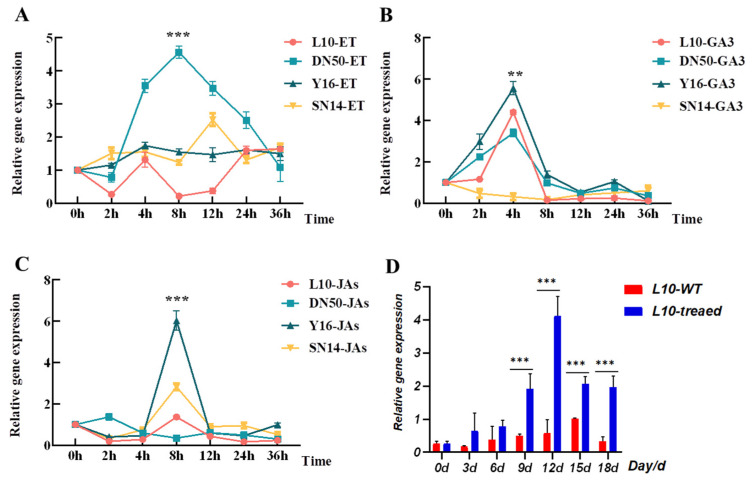
Analysis of *GmPP2C-37like* gene expression pattern in soybean root with different hormone treatments. DN L10 and Y16 are highly resistant to SCN germplasm; DN 50 and SN 14 are highly susceptible to SCN germplasm. (**A**): ET treatment; (**B**): JAs treatment; (**C**): GA3 treatment; dynamic gene expression analysis of extreme resistant materials under SCN stress or non-treatment. (**D**): Expression pattern analysis of the gene in roots of soybean cultivar Dongnong L10 under SCN-stressed and non-stressed conditions at 0–18 d post-treatment; Figure ** significant at *p* < 0.01; *** indicating a very significant difference at *p* < 0.01.

The qRT-PCR results showed that in disease resistance, the expression of *GmPP2C-37like* gene changed significantly under the treatment of JAs, and the results showed that the relative expression increased and then decreased, and reached the maximum value after 12 h of treatment; under the treatment of GA3, the expression of *GmPP2C-37like* gene expression changed significantly in disease-resistant lines Y16 and L10, and reached the maximum value at 4 h of treatment, while it did not change significantly in disease-sensitive line SN14. The expression of the *GmPP2C-37like* gene changed significantly under ET treatment in susceptible DN50 and reached the maximum value at 8h of treatment, while it did not change significantly in susceptible family lines. This indicates that JA can rapidly activate the expression of the disease-resistance candidate gene *GmPP2C-37like* in the disease-resistant lines L10 and Y16 (Figure 4A–C), which means that *GmPP2C-37like* can respond to JA signals in response to stress. Under SCN 3 stress, the relative expression level of *GmPP2C-37like* in DN L10 reaches a peak at 12 d, which is the SCN J_2_ stage and also the stage when soybean is most severely damaged, indicating that this gene is involved in the response to SCN (Figure 4D).

### 2.10. Bioinformatics Analysis and Phylogenetic Tree Analysis of GmPP2C-37like

The coding sequence of the *GmPP2C-37like* gene was analyzed bioinformatically and encoded 401 amino acids with a molecular mass of 97.87 Ku, an amino acid isoelectric point PI = 5.01, and a chemical molecular formula of C_3497_H_5793_N_1203_O_1459_S_307_. The protein signal peptide is non-secretory (Figure S5A); the protein exhibits overall hydrophilicity (Figure S5B); the protein has no transmembrane region (Figure S5C), and there are three spatial configurations in the protein secondary structure (Figure S5D), including α-helix (33.50%), irregular coil (52.50%), extended chain (14.00%), and four structures, with α-helix and irregular coil accounting for the highest proportion. Phosphorylation sites GmPP2C-37like protein exists in 39 potential phosphorylation sites (Supplementary Figure S5E), including 12 threonine (Thr) sites, 25 serine (Ser) sites, and 2 tyrosine (Tyr) sites. The results of phylogenetic tree construction showed that the *GmPP2C-37like* gene has homologs in all crops. The homology comparison with soybean, maize, and *Arabidopsis* plants can be divided into three branches, among which the *GmPP2C-37like* gene has the highest homology with soybean *Glyma.11G222600* gene (Figure S6).

### 2.11. Analysis of Resistance to Soybean Cyst Nematode (SCN)

**Figure 5:**
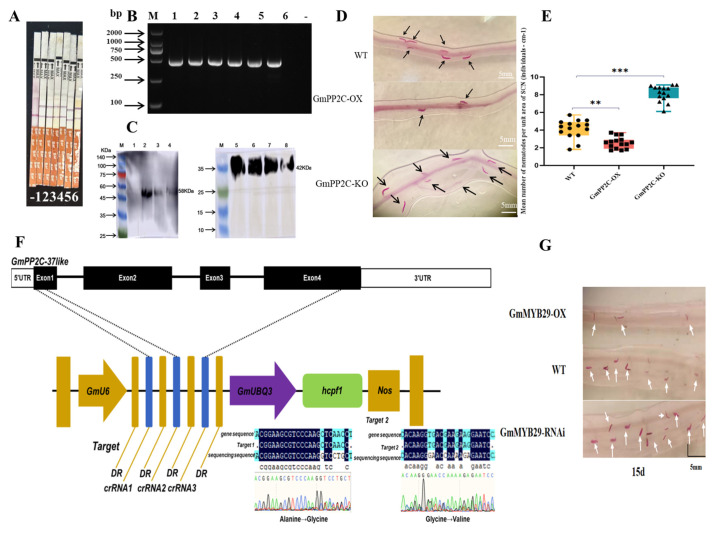
Identification of SCN 3 resistance. (**A**): BAR test strips for determination of soybean hairy roots,-:DN 50 CK, 1–3: Transformed *GmPP2C-37like*-OX root system identification (receptor material DN 50), 4–6: Transformed *GmPP2C-37like*-KO root system identification (receptor material DN 50) (**B**): PCR specific amplification of *BAR* gene in soybean roots, 1–3: *GmPP2C-37like*-OX,4–6: *GmPP2C-37like*-KO; (**C**): M: Colored protein marker; 1: Root protein of soybean cultivar “Dongnong 50” (GFP Protein); 2–4: Protein of overexpressed hairy roots (GFP); 5: Root protein of soybean cultivar “Dongnong 50” (β-Actin4 protein); 6–8: Protein of overexpressed hairy roots (β-Actin4 protein); (**D**): Phenotypic graph of SCN identification; WT: Carmine staining results of lateral roots of wild-type DN50 soybean (after SCN 3 stress); *GmPP2C-OX*: Carmine staining results of lateral roots of *GmPP2C-37Like*-overexpressing soybean; *GmPP2C-KO*: Carmine staining results of lateral roots of *GmPP2C-37Like*-gene-edited soybean. (**E**): Box plot of SCN distribution in *GmPP2C-37like*-OX and wild-type (CK) and *GmPP2C-37like*-KO nematodes. Circle: Distribution per unit area in the roots of WT lines; Square: Distribution per unit area in the roots of OX lines; Triangle: Distribution per unit area in the roots of KO lines. (**F**): *GmPP2C-37-like*-gene-editing plasmid basic framework and gene target sequence editing information. Green: Adenine (A); Black: Guanine (G); Red: Thymine (T); Blue: Cytosine (C). (**G**): Phenotypic graph of SCN identification; *GmMYB29-OX*: Carmine staining results of lateral roots of *GmMYB29*-overexpressing soybean; *GmMYB29*-RNAi: Carmine staining results of lateral roots of *GmMYB29*-RNAi-mediated soybean. Figure ** significant at *p* < 0.01; *** indicating a very significant difference at *p* < 0.01. Arrow: SCN infecting soybean roots after fuchsin acid staining.

At the protein expression level, the Bar test strip results showed that the OE-*GmPP2C-37like* and KO-*GmPP2C-37like* soybean hairy roots were identified as transgenic, while the wild-type (WT) soybean hairy roots were non-transgenic (Figure 5A). In addition, at the DNA level, specific PCR amplification was performed on the hairy root DNA using Bar-F/R primers (Table S10). At the protein level, distinct protein bands of the internal reference protein β-Actin4 were observed in both the wild-type (WT, Lane 5) and overexpression (OE, Lanes 6–8) samples. In contrast, the GFP fusion protein showed distinct protein bands in the OE samples (Lanes 2–4) but no detectable bands in the WT sample (Figure 5C). The results indicated that the corresponding soybean hairy roots were transgenic, as a clear band was observed at 480 bp in 2% agarose gel electrophoresis, confirming the successful acquisition of transgenic soybean hairy roots (Figure 5B). Gene editing results showed that target 1 underwent a base mutation at 162 bp in the first exon, with GCT encoding alanine and GGT encoding glycine, resulting in a non-synonymous mutation. Target 2 exhibits a base mutation at the 224 bp position in the first exon, where GGA encodes glycine and GTG encodes valine, resulting in a non-synonymous mutation (Figure 5F). A statistically significant difference was observed in the number of nematodes in the root systems of transgenic and wild-type roots using the acid carmine staining method. The results showed that the average number of nematodes per unit area in OE was 2.6/cm, while the average number of nematodes per unit area in KO was 8.1/cm. The average number of nematodes in the lateral roots of the wild-type root system was 4.1/cm. There was a greater variability in the number of nematodes in the hairy roots of *GmPP2C-37like* overexpressing transgenic soybean compared with CK, which can prove that *GmPP2C-37like* gene has obvious resistance to SCN disease (Figure 5D,E, Table S9). Compared with the control group (CK), the number of nematodes in the root hairs of *GmPP2C-37like* gene-edited transgenic soybeans showed greater variability, indicating that the *GmPP2C-37like* gene has a significant positive regulatory effect on SCN disease (Figure 8).

Fifteen positive roots were obtained from transformations with the *GmMYB29*-pCAMBIA3300 vector and GmMYB29-RNAi vector. These transgenic roots were inoculated with SCN 3. After 15 days, the roots were stained using the acid carmine staining method and observed/counted under an optical microscope. The number of cyst nematodes in *GmMYB29*-overexpressing roots was lower than that in wild-type (WT) roots, while the number of cyst nematodes in roots with *GmMYB29* expression interfered by RNAi (*GmMYB29*-RNAi roots) was higher than that in WT roots. Additionally, the developmental stage of cyst nematodes in GmMYB29-overexpressing roots was later than that in *GmMYB29*-RNAi roots and WT roots. Paired sample *t*-tests were performed between *GmMYB29*-overexpressing roots and empty vector-transformed roots, as well as between *GmMYB29*-RNAi roots and empty vector-transformed roots (Figure 5G, Tables S13 and S14). The results showed that the *p*-values of the two-tailed *t*-tests were all less than 0.01, indicating that *GmMYB29* can enhance soybean.

### 2.12. Subcellular Localization of GmPP2C-37like: Observation via Laser Confocal Microscopy in Agrobacterium-Infiltrated Plants

**Figure 6:**
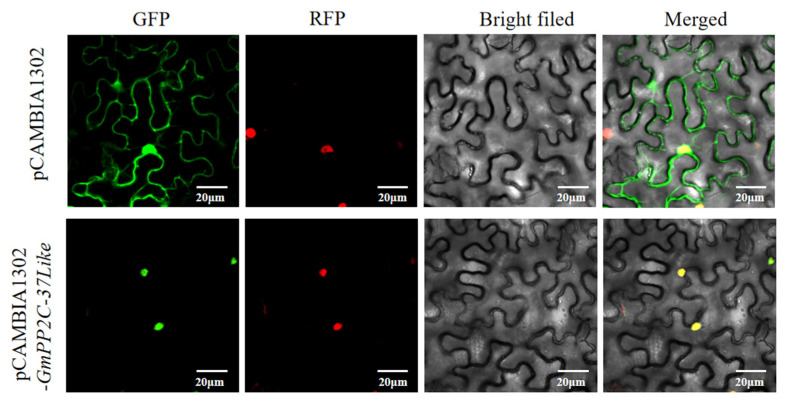
Results of subcellular localization of the *GmPP2C-37like* gene expression. GFP (green fluorescent protein); RFP (red fluorescent protein), nuclear marker; bright filed: without fluorescent irradiation; merged: multiple occasions.

The successfully constructed pCAMBIA1302-*GmPP2C-37like* plasmid was successfully transformed into the Agrobacterium recipient cell GV3101 (psoup-p19). The *Agrobacterium* with the empty vector pCAMBIA1302-GFP was used as the control group. Laser confocal microscopy was performed to observe the distribution of GFP (green fluorescent protein) in the plants. The results showed that in pCAMBIA1302-GFP empty vector, there was a certain amount of expression in the cell membrane and nucleus as well as in the cytoplasm. pCAMBIA1302-*GmPP2C-37like* was the brightest in the nucleus of the plant, so the protein encoded by the *GmPP2C-37like* gene is mainly expressed in the nucleus belonging to the nucleus-localized proteins (Figure 6).

### 2.13. Yeast Two-Hybrid Library Screening Analysis

**Figure 7:**
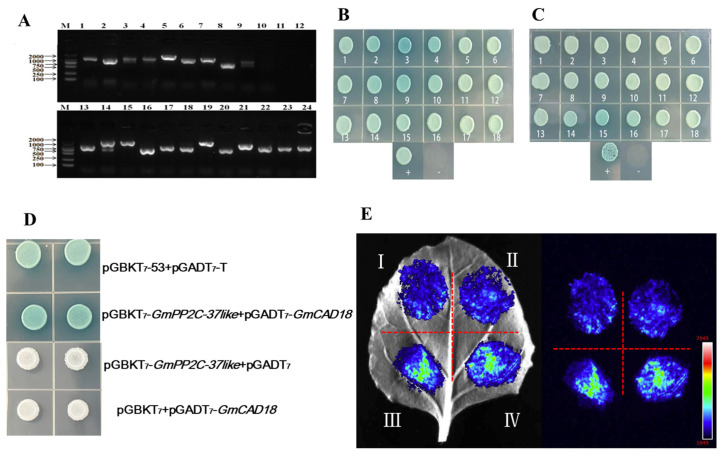
Yeast two-hybrid library screening. (**A**): Yeast liquid PCR identification results; (**B**): SD/-Trp/-Leu/-His/X-α-Gal solid plate colony growth 1–18, -: Negative control, +: Positive control; (**C**): SD/-Ade/-Trp/-Leu/-His/X-α-Gal solid plate colony growth 1–18, -: Negative control, +: Positive control. (**D**): Yeast one-to-one interaction identification, I: pGBKT_7_-53 + pGADT_7_-T; II: pGADT_7_-*GmPP2C-37like* + pGBKT_7_-*GmCAD18*; III: pGBKT_7_-*GmPP2C-37like* + pGADT_7_; IV: pGBKT_7_ + pGADT_7_-*GmCAD18*; (**E**): I: p1300-nLUC-*GmPP2C-37like* + p1300-cLUC; II: p1300-nLUC+ p1300-cLUC-*GmCAD18*; III: p1300-nLUC-*GmPP2C-37like* + p1300-cLUC-*GmCAD18*, IV: p1300-nLUC-T + p1300-cLUC-p53.

According to the physical map of pGBKT_7_, *ECO*R I and *Bam*H I were designed (Figure S7A), and the plasmid was linearized. The result showed that the gel speed of the linearized fragment of the loss of super-helical structure was greater than the speed of the plasmid, and the plasmid was linearized successfully (Figure S7C). The coding sequence (CDS) of the *GmPP2C-37like* gene was homologously recombined with the linearized pGBKT_7_ vector. The bacterial solution was spread onto SD/-Trp dropout solid medium, resulting in the successful growth of single yeast colonies (Figure S7B). After identification by specific PCR and successful sequencing alignment, the construct was successfully transformed into the Y_2_H yeast strain. After resuscitation and mixing the co-culture with AD library bacterial solution, the growth of blue single spot was picked in SD/-Trp/-Leu/-His liquid medium and amplified by specific PCR using universal primer AD-F/R. Blast sequence comparison of the sequencing results using Phytozome V_13_ screened a total of 18 candidate genes (Figure 7A–C, Table 1).

The yeast of the positive control group (pGBKT_7_-53 + pGADT_7_-T) formed blue colonies on the selective medium, and the reporter gene was successfully activated, indicating that the experimental system was reliable. The yeast of the combination to be verified (pGBKT_7_-*GmPP2C-37like* + pGADT_7_-*GmCAD18)* also formed blue colonies, suggesting that *GmPP2C-37like* interacts with *GmC_4_H* in yeast cells. The yeast of the negative control combinations (pGBKT_7_-*GmPP2C-37like* + pGADT_7_ and pGBKT_7_ + pGADT_7_-*GmCAD18*) only formed white colonies with no reporter gene activation, demonstrating the specificity of the aforementioned interaction (Figure 7D). In the dual-luciferase complementation assay, only low blue signals were detected in the negative control combinations I(p1300-nLUC-*GmPP2C-37like* + p1300-cLUC) and II(p1300-nLUC + p1300-cLUC-*GmCAD18*), indicating that there was no non-specific complementation between *GmPP2C-37like* or *GmC_4_H* and the luciferase fragments. The combination to be verified III (p1300-nLUC-*GmPP2C-37like* + p1300-cLUC-*GmCAD18*) exhibited high green-to-yellow signals, which suggested that the nLUC and cLUC fragments of luciferase were successfully complemented due to the interaction between *GmPP2C-37like* and *GmCAD18*, confirming that the two proteins interact in plant leaf cells. Effective fluorescent signals were detected in the positive control combination IV (p1300-nLUC-T + p1300-cLUC-p53: a pair of known interacting proteins), verifying the validity of the experimental system (Figure 7E).

## 3. Discussion

Based on the diverse characteristics of SCN populations, Fujita et al., in 1934, classified the SCN into many subspecies [20]. In 1957, Ross et al. found that nematode populations may differ between physiological microspecies [21]. In 1962, Ross reported for the first time that SCN populations in different regions differed in their susceptibility to soybean [22], and in 1970, Golden used physiological microspecies (Race) to classify SCN into physiological microspecies with different pathogenicity [23]. Pickett, PI548402 (Peking), PI88788, and PI90763 soybeans were identified as resistant hosts, with Lee68 as a susceptible host. The distribution of SCN in China is mainly in the Heilongjiang, Jilin, Liaoning, Inner Mongolia, etc., provinces [24,25,26]. SCN in the United States is mainly distributed in 27 states, including Alabama, Arkansas, and Arizona. [27,28,29,30,31,32,33,34,35]. Brazilian SCN is mainly distributed in Bahia, Goias, Maranhão, São Paulo, Tocantins, and Rio Grande do Sul in a denser distribution [36]. There are also reports of nematode occurrence in Hokkaido, Kyushu, and Honshu in Japan, and in North Korea, South Korea, and the United Kingdom. It can be seen that SCN is widely distributed worldwide and is a highly destructive soil-borne disease.

At present, the preventive measures for SCN mainly use reasonable crop rotation, chemical pesticides, biological control, and resistant soybean varieties. The crop has a certain immune response of its own, and proper fertilization will also increase the enhancement of crop resistance to SCN. Planting resistant soybean germplasm results in SCN being difficult to reproduce and survive in the root system of the resistant germplasm; only a small proportion of cyst nematodes can grow, and SCN-resistant soybean can effectively reduce nematode density per unit area [37]. However, the disadvantage is that the long-term planting of a single source of resistance will lead to a small number of viable cysts continue to multiply, resulting in soybean from “SCN-resistant” to “SCN-sensitive”, resulting in the loss of resistance to the source of resistance. Therefore, rotating the source of resistance is also a very necessary means and method. In 1957, Ross and Brim identified 2800 soybean materials planted into severe SCN-diseased soils for disease resistance, and the identification results showed that Ilsoy, Peking, PI89920, PI79693, PI90763, PI209332, PI84751, and PI88788 were highly resistant to SCN materials [21,38,39]. In recent years, SCN-resistant varieties such as SCN-resistant varieties 1–13, Qingfeng 1, Zhonghuang 12, Zhonghuang 13, Zhonghuang 17, Zhongpin 03-5373, Nenfeng 14–15, 20, 6092 DN-L10, etc., [38,39,40], have been widely disseminated, and they have a good effect of nematode resistance. Crops such as maize and oat SCN cannot grow and reproduce in the root system, and crop rotation for several years can reduce SCN damage to soybeans to some extent. Accompanied by the increase in the number of years of crop rotation, the density of nematodes per unit area will be significantly reduced [41,42,43,44]. The wheat–barley–corn–soybean rotation can effectively control the development of cyst nematodes in the soil [45]. Some vegetable root systems can produce substances or predatory relationships that are toxic to SCN, e.g., the root system of marigolds produces toxic substances that reduce the unit density of SCN [37]. Peas can stimulate the development of an SCN, leading to premature maturation and death of the SCN, which is unable to adapt to the survival environment [46]. The crop rotation approach is a dynamic balancing act that does not fundamentally eliminate SCN but only reduces the risk of SCN to soybeans. Chemical pesticide use is also a popular means to control SCN. Chemical pesticide methods can be effective in the short term, but SCN numbers may spike when soybeans are harvested. Therefore, it is important to pay attention to issues such as the strength and safety of use. SCN can be effectively controlled by using biological control methods, which utilize natural enemies of SCN to suppress the survival of SCN and nematode density per unit area. At present, the biological control of SCN has a wide variety of bacteria and is easy to isolate, so the prospect of application is also very promising. Biocontrol organisms have been reported as *Paecilomyces lilacinus*, *Fusarium* spp. and *Verticillium chlamydosporia* [47]. *Paecilomyces* and *lilacinus Verticillium chlamydosporia* have been made into biopesticides and applied in field production [48,49].

Gene expression analysis revealed that *GmPP2C-37like* has genotype-specific responses to JA, GA3, and ET—hormones linked to plant biotic stress. In SCN-resistant lines (DN L10, Y16), JA induced its expression peaking at 12 h (no change in susceptible SN14); GA3 triggered a 4 h peak in resistant lines (no response in susceptible ones). ET led to an 8 h expression peak in susceptible DN50, indicating that hormone-induced *GmPP2C-37like* activation depends on soybean genetic background. This hormone responsiveness aligns with the known role of *PP2C* genes in hormone signaling. Subfamily A of *PP2C* genes negatively regulates abscisic acid (ABA) signaling, while subfamily B participates in MAPK pathway regulation. In this study, *GmPP2C-37like*’s response to JA is particularly notable: JA is a key regulator of plant defense against nematodes, as it mediates the synthesis of defensive compounds and the reinforcement of physical barriers. The peak of *GmPP2C-37like* expression at 12 h under JA treatment coincides with the J_2_ stage of SCN—the stage when nematodes penetrate soybean roots and form syncytia, causing the most severe damage. *GmPP2C-37like* shows genotype-specific responses to GA3 (a plant biotic stress-related hormone). Its expression peaked at 4 h in SCN-resistant lines (DN L10, Y16) under GA3 treatment, with no obvious response in susceptible lines. This temporal alignment suggests that *GmPP2C-37like* is activated by JA GA3 signals to counteract SCN infection at the critical stage of root colonization, highlighting its role as a hormone-responsive mediator of resistance.

We searched for *GmPP2C-37like* interactions and obtained 18 candidate genes through the screening of the AD yeast library. In which the *Glyma.08G112300* gene is attributed to one of the AAI_LTSS superfamily members. AAI_LTSS (alpha-amylase inhibitors (AAI), lipid transfer (LT), and seed storage (SS)) is a protein family unique to higher plants that includes cereal-type alpha-amylase inhibitors, lipid transfer proteins, seed storage proteins, and similar proteins. Proteins in this family are known to play important roles in defending plants from insects and pathogens, lipid transport between intracellular membranes, and nutrient storage. *Glyma.20G148400* belongs to S vicilin seed storage globulin and N-terminal cupin domain. Proteins in this family have a variety of functions, including enzymes associated with sucrose binding, dehydration, resistance to microbes, and oxidative stress. The *Glyma.16G155500* gene belongs to one of the CASP family members, and it has been reported in the literature to demonstrate that CASP is the first molecular factor demonstrated to establish plasma membrane and extracellular diffusion barriers in plants and represents a novel approach to epithelial barrier formation in eukaryotes. The role of CASP in constructing and localizing cell wall repair was demonstrated [50]; it is interesting to note that SCN-infested soybean root system in J_2_ period breaks down cellulase and pectinase and forms syncytia with soybean root system and parasitises in the root system. And there is some function in cell wall modification of *Glyma.16G155500* gene in CASP family, and it is speculated that the gene may be involved in soybean cyst nematode disease. Eighteen candidate genes were screened and analyzed for gene expression using the public database Phytozome. The results showed that the genes *Glyma.08G206000, Glyma.11G013200,* and *Glyma.01G230200* had the highest relative expression, 71.57, in the plant root system, and it was hypothesized that these genes might respond to SCN disease or adaptation (through gene expression optimization, it enhances tolerance to SCN and even reduces disease impact, with a greater emphasis on long-term and stable characteristics.) (Figure S8). Future studies will focus on the “adaptation mechanism.” Genes stably expressed at 14 d, stress resistance pathway-enriched, and soybean SCN resistance-specific will be screened. Combined with differential expression levels and protein–protein interactions, 8–10 core adaptive genes will be identified. Their effects on soybean SCN adaptation will be verified via gene editing/overexpression, with stress-resistant phenotype correlations clarified and pathway node functions localized. Using these core genes as hubs, upstream/downstream interacting genes will be connected to form a complete pathway, with efficiency optimized and redundancies eliminated. Finally, pathway-related genes will be applied to marker-assisted breeding, the complete pathway introduced into susceptible soybeans via transgenesis, and multi-module adaptive pathways will be integrated to establish a synergistic stress resistance network.

**Figure 8:**
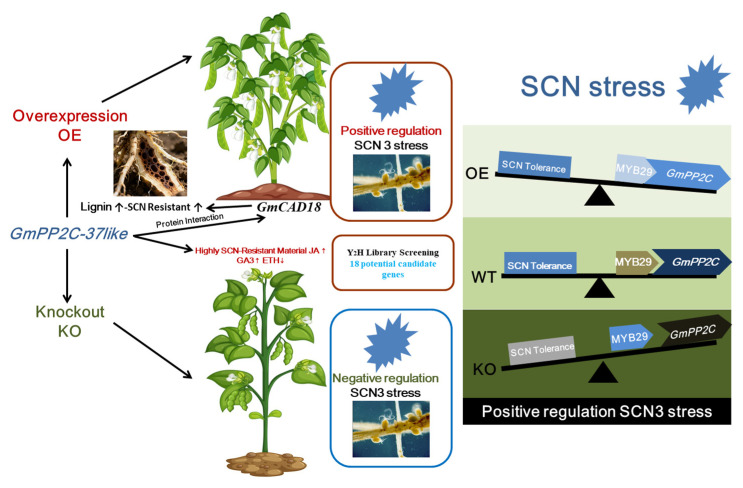
Mechanism diagram of participating in SCN stress.

This study hypothesizes that the *GmPP2C-37like* gene directly promotes the formation of defensive structures. By positively regulating callose deposition, it forms a physical barrier to restrict the progression of SCN disease. Specifically, this gene may enhance the expression of callose synthase genes, thereby strengthening cell wall reinforcement and impeding the movement and nutrient uptake of SCN in soybean root tissues. The *GmCAD18* gene was screened from the yeast library. This gene is involved in lignin synthesis and serves as one of the key enzymes in the lignin biosynthetic pathway. It can catalyze a variety of cinnamaldehydes (such as *p*-coumaraldehyde, sinapaldehyde, and coniferaldehyde), and thus affects plant mechanical strength, cell wall integrity, and resistance to pathogens. Lignin accumulation can enhance the physical barrier function of plant cell walls against pathogens, helping plants resist pathogen invasion (Figure 8). The results will provide an important foundation for the subsequent synergistic regulation of SCN molecular mechanisms.

## 4. Materials and Methods

### 4.1. Materials

#### 4.1.1. Plant Material

The plant materials used in this study mainly included soybean germplasm DN 50 (SCN 3-sensitive type), DN L10 (SCN 3-resistant type), SN14 (SCN 3-sensitive type), and Y16 (SCN 3-resistant type), and all soybean germplasm resources were planted in 2023 in Harbin City, Heilongjiang Province (45°45′16.2″ N, 126°54′39.6″ E) at Xiangyang Farm. The length of rows was 2 m, the width of rows was 0.65 m, the spacing between plants was 0.06 m, and there were three biological replications. *Nicotiana benthamiana* was planted in a thermostatic incubator at a temperature setting of 28 °C. The T_3_-*GmMYB29* overexpressing transgenic soybean lines were obtained by infecting soybean cotyledonary nodes with *Agrobacterium* strain EH105 (expression vector pCAMBIA3300-35S-GFP-*GmMYB29*). From 2022 to 2023, these lines were propagated through indoor and pot-based generation acceleration and were prepared for CHIP-Seq sequencing. T_3_-*GmMYB29* overexpressing transgenic soybean lines (with DN50 as the recipient, named OE_MYB_SCN3) and wild-type (WT) plants (DN50, named DN50_SCN3) were germinated to the V_2_ stage using vermiculite. After the seedlings were transplanted and subjected to nematode stress for 14 d, the plant root tissues were collected for RNA-Seq sequencing.

#### 4.1.2. Diseased Soil Material

SCN 3 originated from the diseased soil experimental site in Daqing Anda District (46°23′24.000″ N, 125°21′36.000″ E) and was identified by the Riggs and Schmitt model [5]. SCN is isolated from the soil, purified, and preserved by the Key Laboratory of Northeast Soybean Biology and Genetic Breeding, Ministry of Agriculture and Rural Affairs, Northeast Agricultural University.

### 4.2. Methods

#### 4.2.1. CHIP-Seq and RNA-Seq Sequencing of GmMYB29 Strain

Fresh roots of 2 g from the *GmMYB29* transgenic T_3_ generation lines were collected, in vitro cross-linking reaction, tissue lysis with DNA shearing, protein/DNA immunoprecipitation, cross-linking DNA reversal/DNA purification, library construction, and sequencing on Illumina platform. The antibody tag protein selected in this study is GFP (green fluorescent protein). Sequencing of purified DNA after immunoprecipitation was performed using second-generation high-throughput sequencing (HTS) Illumina/Solexa, Roche/454, SOLiD (ABI), and Ion Torrent. Sequencing results were subjected to quality control to search for transcription factor-encoding proteins bound to Peak Calling. CHIP-Seq data were annotated and visualized using the CHIP seeker package v1.45.0 for the R language.

High-quality mRNA is enriched via Oligo (dT)-containing magnetic beads and then fragmented into short segments by fragment buffer. Using these as templates, double-stranded cDNA is synthesized, which, after repair, A-tailing, and sequencing adapter ligation, undergoes fragment size selection via AMPure XP beads. PCR products are purified with AMPure XP beads, finally yielding a strand-specific cDNA library for HiSeq sequencing. The above sequencing and analysis were performed by Boyue Zhihe Biotechnology Co., Ltd. (Wuhan, China).

#### 4.2.2. KEGG and GO Enrichment

Transcription factors were selected in conjunction with Peak Calling for distance TSS −2000 bp candidate genes, which were analyzed using Omicshare (https://www.omicshare.com/tools/ accessed on 1 May 2024) for KEGG (Kyoto Encyclopedia of Genes and Genomes) and GO (Gene Ontology) enrichment, and the results were visualized and analyzed. Based on the CHIP-Seq sequencing results and distance from TSS (transcription start site) −2000 bp, all potential site candidate genes were collated, and the genes were visualized using KEGG enrichment analysis in the Kidio Bioinformatics Platform (https://www.omicshare.com/ accessed on 1 May 2024), and reference genome *Williams 82*_V2.0_ was selected. Visualization of potential genes from CHIP-Seq and RNA-Seq results was performed by using the KEGG database.

#### 4.2.3. RNA-Seq and CHIP-Seq Joint Analysis

Root transcriptome sequencing analysis was performed on SCN 3-stressed overexpression lines and WT lines for 15 d. Differential multiples were screened using thresholds of |log_2(Fold change)_| > 1 and *p* < 0.05. KEGG visualization analysis was conducted through the integration of RNA-Seq differentially expressed genes and CHIP-Seq data.

#### 4.2.4. Dual-Luciferase Assay

Nanodrop measured A_260_/A_280_ results showed that the measured concentration was 1.8–2.0; the results showed that the purity of DNA extracted from the root system of DN L10 was good (Figure S3A). The results showed that the vector was amplified by PCR using specific primers and detected by gel electrophoresis, and the bands were around 2000 bp, and the vector was successfully constructed (Figure S3B and Figure 3C).

The pGreenII 0800-LUC and pCAMBIA3300 plasmid linearization systems, along with the homologous recombination systems, were each prepared in a total volume of 20 μL (Tables S1 and S2). The recombinant vector pCAMBIA3300-35S::*GmMYB29* GV3101 (pSoup-p19) was subsequently injected into tobacco leaves following co-cultivation with pGreenII-0800-LUC 1-LUC 12 GV3101 (pSoup-p19). Individual pGreenII-0800-LUC 1-LUC 12 bacterial suspensions served as negative controls, and these suspensions were injected into *Nicotiana benthamiana* leaves for a duration of 4 h in the absence of light. After being incubated in the dark for 60 h, the leaf surfaces were treated with 1 × D-Luciferin Sodium Salt D (Thermo Fisher Scientific, Waltham, MA, USA). Fluorescence imaging was performed using a fully automated chemiluminescence imaging analysis system (Tanon 5200, Shanghai, China).

#### 4.2.5. Promoter DNA Truncation Analysis

The promoter DNA of *GmPP2C-37like* was truncated into four fragments (I–IV), corresponding to regions −397, −1159, −1572, and −1852 bp upstream of the TSS (transcription start site), respectively. These four subcloned promoter fragments were then individually recombined with the pGreenII-0800 vector via homologous recombination. Subsequently, *Nicotiana benthamiana* leaves were injected with *Agrobacterium* harboring pCAMBIA3300-35S::*GmMYB29* and *Agrobacterium* harboring pGreenII-0800-I-IV following co-cultivation.

#### 4.2.6. Yeast One-Hybrid Study

The *GmPP2C-37like* promoter, spanning −2000 bp, was homologously recombined with linearized pAbAi using the ClonExpress II One Step Cloning Kit (Vazyme, Nanjing, Jiangsu, China) (Table S3). Additionally, *GmMYB29* coding sequences (CDs) were homologously recombined with linearized pGAD-T_7_ (Table S4). The plasmids pAbAi-*ProGmPP2C-37like* and pGAD-T_7_-*GmMYB29* were co-transformed into Y_1_HGold yeast. The yeast was then spread on SD/-Leu/ABA* solid medium and incubated at 30 °C for 3–5 d to allow for the growth of positive single colonies. The specific methodology followed the instructions provided in the Y_1_ HGold yeast transformation manual. On plates containing SD-Leu + Aba^200^ and SD-Leu + Aba^600^, the yeast suspensions were diluted 1×, 10×, and 100×, respectively. Then, 10 µL of the diluted yeast solutions pGAD-T_7_-*GmMYB29*/ pAbAi-*ProGmPP2C-37like* and pGAD-T_7_-*GmMYB29* were dropped onto the AbAi (Aureobasidin A)-containing solid plates using a pipette.

#### 4.2.7. GUS Staining Analysis

The GUS staining method used was the GUS staining kit (SL7160, Coolaber, Beijing, China). *Agrobacterium* containing pCAMBIA3300-*GmMYB29* and pBI121-GUS-*proGmPP2C-37like Agrobacterium* was cultured overnight, and the tobacco buffer was prepared (Table S5). After centrifugation of the samples, the samples were utilized to adjust the bacteriophage OD_600_ to 0.8 using tobacco buffer, and incubated for 3 h at 25 °C. The control group was *Agrobacterium* pBI121-GUS-*proGmPP2C-37like* only, and the operation method was the same as that of the test group. The test group and CK were injected with *Agrobacterium* infiltration buffer into tobacco leaves and stained and decolorized using GUS staining kit operation.

#### 4.2.8. Analysis of GmPP2C-37like Gene Expression Pattern

In plant hormones, ET, Jas, and GA3 are important signaling molecules in the signal transduction pathway of plant defense response. The extreme SCN-resistant materials Y16 and DN L10 and the susceptible SCN materials DN 50 and SN 14 were treated with ET (500 μmol/L), Jas (50 μmol/L), and GA_3_ (50 μmol/L) [4,51,52]. Soybean roots treated for 0 h, 2 h, 4 h, 8 h, 12 h, 24 h, and 36 h were extracted, and root RNA was extracted using the Trizol method (Thermo Fisher Scientific, USA). Reverse transcription of cDNA from RNA was performed by using PrimeScript™ RT reagent Kit (Perfect Real Time) (Takara, USA) (refer to the instructions for the procedure) (Table S10). *GmActin 4* was selected as the internal reference gene for fluorescence quantitative PCR. qRT-PCR was performed by SYBR method after treatment with SCN 3 stress in resistant soybean. The formula was calculated with reference to ΔCt = Ct_target gene_ − Ct_internal reference gene_, ΔΔCt = ΔCt_A sample_ − ΔCt_B sample_, and relative gene expression = 2^−ΔΔCt^ to obtain the relative expression of genes, and the data were statistically analyzed and visualized using GraphPad Prism 10.

#### 4.2.9. The Construction of Vectors Related to GmPP2C-37like and GmMYB29

The coding sequence (CDS) of *GmPP2C-37like* was homologously recombined with linearized pCAMBIA3300, using 20 μL each of the pCAMBIA3300 linearization system and the homologous recombination system. The basic backbone for the gene-editing vector construction is pCRISPR-Cas12 (Cpf1). The homologous recombination technique is used to ligate the Cpf1 Acidaminococcus vector, which has been double digested with *Ahd* I/*Ade* I, with the PCR-purified product of the target sequence (Table S10).

Linearization of pCAMBIA1302 using *NOC* I showed that there was a certain electrophoretic distance between the linearized fragment and the plasmid, so it was determined that the pCAMBIA1302 plasmid *NOC* I cut was complete (Figure S4B). Linearization of pCAMBIA3300 using *Hin*d III showed that there was a certain electrophoretic distance between the linearized fragment and the plasmid, so it was determined that the pCAMBIA300 plasmid *Hin*d III enzyme was completely cut (Figure S4A). The exon sequences were cloned for overexpression and subcellular localization of *GmPP2C-37like* exon sequences (Figure S4C). The homologous recombination of the pCAMBIA3300*-GmPP2C-37like* and pCAMBIA1302-*GmPP2C-37like* vectors was successful (Figure S4D).

Primers *GmMYB29*-3300-F/R were designed to amplify the target fragment from DN L10, followed by sequencing (Table S10). The *GmMYB29*-pCAMBIA3300 recombinant vector was transformed into *Agrobacterium rhizogenes* K599. For constructing the RNAi vector, forward amplification primers *GmMYB29*-RNAi-1F/R and reverse amplification primers *GmMYB29*-RNAi-2F/R were designed (Table S10). The amplified products, with a size of 400 bp, were ligated with the pFGC5941 vector, and the recombinant vectors were transformed into *Agrobacterium rhizogenes* K599.

#### 4.2.10. Bioinformatics Analysis and Phylogenetic Tree of GmPP2C-37like

Protein signal peptide prediction analysis was performed by using the online tool Signalp (https://novopro.cn/tools/signalp accessed on 1 May 2024). Protein hydrophilicity and hydrophobicity analyses were performed using Novopro 3.0 (https://www.novopro.cn/tools/protein-hydrophilicity-plot.html accessed on 1 May 2024). Protein transmembrane region was performed using (https://www.novopro.cn/tools/tmhmm.html accessed on 1 May 2024). The protein structure was analyzed using NPS (https://npsa.lyon.inserm.fr/cgi-bin/npsa_automat.pl?page=/NPSA/npsa_sopma.html accessed on 1 May 2024) and SWISS-MODE (https://swissmodel.expasy.org/ accessed on 1 May 2024). Phosphorylation site analysis was performed bioinformatically using NetPhos v. 3.1 (https://services.healthtech.dtu.dk/services/NetPhos-3.1/ accessed on 1 May 2024).

The homologous proteins of the *GmPP2C-37like* gene in soybean, Arabidopsis, and other plants were organized into Fasta format, and the homologous proteins were compared by using Mega X. The process included loading, Align protein, phylogenetic analysis, Neighbor-Joining (N-J) method, Bootstrap times of 1000, and the comparison of homologous proteins using Mega X. The homologous proteins of *GmPP2C-37like* gene in soybean, *Arabidopsis* and other plants were organized into Fasta format. The procedure was protein sequence loading, Align protein, phylogenetic analysis, comparison by Neighbor-Joining (N-J) method, Bootstrap times of 1000, and construction of evolutionary tree; Chiplot tool (https://www.Chiplot.online/tvbot.html accessed on 1 May 2024) was used to beautify the phylogenetic tree.

#### 4.2.11. Identification of Agrobacterium K599 Transformed Soybean Hairy Roots and SCN

Dongnong 50, a susceptible soybean variety, was cultivated in vermiculite. The primary root was subjected to treatment with *Agrobacterium* K599 bacterial solution containing pCAMBIA3300-*GmPP2C-37like* and CRISPR-Cas12 (Cpf1)*-GmPP2C-37like* to induce a wound at the junction of the soybean root system and the stem. Some hairy roots Bar test strips were taken for detection. Subsequently, these plants were transferred to pots containing SCN 3, with an average of 200 SCN eggs selected per pot to enhance the unit density of SCN in the soil. After 14 d, the roots were extracted from the infested soil, and the transgenic hairy roots were identified through magenta staining, which quantified the number of nematodes present in the root system. J_2_ SCN were observed using a 20× microscope to assess the number of SCN per unit area, and the data were statistically analyzed and visualized using IBM SPSS 29.0 and GraphPad Prism 10. The identification method for hairy roots of OX-*GmMYB29* and RNAi-*GmMYB29* soybeans is also consistent with the above-mentioned method.

#### 4.2.12. Subcellular Localization of GmPP2C-37like

*GmPP2C-37like* CDs were used for homologous recombination with linearized pCAMBIA1302: 20 μL for the pCAMBIA1302 linearization system and 20 μL for the homologous recombination system (Table S6). *Nicotiana benthamiana* growth to 28 d was followed by *Agrobacterium* GV3101 (Psoup-p19) injection into the lower epidermis of tobacco and was treated for 24 h under dark conditions. Plants were exposed to strong light 1 h before infestation, and the injection parts were the top downward third out of the young leaves. The fungus was resuspended with tobacco transformation solution, and the OD_600_ was adjusted to 0.8 by adding tobacco resuspension solution and was incubated at a constant temperature for 5 h. After tobacco injection, the fungus was treated with darkness for 12 h-light for 24 h and observed and photographed under a laser confocal microscope (Leica, Wetzlar, Germany).

#### 4.2.13. Yeast Two-Hybrid Library Screen

The pGBKT_7_ linearization system and homologous recombination system are detailed in Table S7. The recombinant plasmid pGBKT_7_-*GmPP2C-37like* was transformed into Y_2_Hgold competent cells and subsequently spread on SD/-Trp plates. Mix 1 mL AD yeast library solution + 4 mL pGBKT_7_-*GmPP2C-37like* bacterial solution + 45 mL 2 × YPDA liquid + 50 μg/mL Kanamycin Sulfate in a 1 L conical flask and incubate at 30 °C, 50 rpm for 24 h. Transfer the bacterial solution to a sterile 50 mL centrifuge tube, centrifuge at 4000 rpm for 10 min, discard the supernatant, and resuspend with 50 mL 0.5 × YPDA liquid. After another centrifugation at 4000 rpm for 10 min, resuspend cells with 10 mL 0.5 × YPDA containing 50 μg/mL kana. Spread 100 μL of this dilution onto SD/-Leu/-Trp/-His/X-α-Gal solid plates (55 plates total) and incubate at 30 °C for 5 d. Pick blue single colonies into 10 mL TDO (SD/-Leu/-Trp/-His) liquid medium and incubate at 30 °C for 48 h. Perform specific PCR on the yeast liquid using universal primer AD-F/R, run the PCR product on 2% agarose gel electrophoresis, purify the target band, and sequence it. Analyze sequencing results with NCBI’s BLAST tool v2.16.0 to identify candidate genes via gene similarity and functional annotation. To further identify candidate genes that potentially interact with *GmPP2C-37like*, dual-luciferase complementation assay and yeast two-hybrid assay were employed to confirm the gene interaction relationships.

## 5. Conclusions

In summary, this study demonstrates that the transcription factor *GmMYB29* directly binds to the critical region spanning −1159 to −1572 bp in the promoter of its downstream gene *GmPP2C-37like*, thereby promoting the latter’s transcription. *GmPP2C-37like* responds to hormone signals such as jasmonic acid and positively regulates soybean resistance to soybean cyst nematode (SCN) race 3: it interacts with proteins like *GmCAD18* to modulate lignin biosynthesis, strengthening cell wall defense. Consistent with this regulatory role, overexpression of *GmMYB29* significantly reduces SCN numbers in soybean roots, while gene editing of *GmMYB29* impairs resistance. Collectively, these findings confirm that the *GmMYB29-GmPP2C-37like* module serves as a core regulatory axis in soybean’s defense against SCN race 3. Future research could explore how this module integrates with other hormone signaling pathways (e.g., salicylic acid or ethylene) to fine-tune SCN resistance and investigate whether manipulating *GmMYB29* or *GmPP2C-37like* expression through biotechnological approaches can yield SCN-resistant soybean varieties with stable agronomic traits. Additionally, identifying upstream regulators of *GmMYB29* or other downstream targets of *GmPP2C-37like* may further unravel the complex regulatory network underlying nematode defense.

## Figures and Tables

**Table 1:** Screening for interaction-related gene information.

Gene ID	Annotation	Start	End	Functional Annotation
*Glyma.13G128900*	AT4G28730.1	24,209,750	24,212,616	Glutaredoxin family protein
*Glyma.05G114900*	AT1G07930.1	30,481,777	30,484,482	GTP binding Elongation factor Tu family protein
*Glyma.14G117700*	/	15,530,056	15,530,697	/
*Glyma.20G090100*	AT5G26710.1	33,247,170	33,252,176	Glutamyl/glutaminyl-tRNA synthetase, class Ic
*Glyma.08G112300*	AT1G55260.1	8,648,286	8,649,896	Bifunctional inhibitor/lipid-transfer protein/seed storage 2S albumin superfamily protein
*Glyma.15G136100*	AT1G30630.1	10,998,218	11,001,584	Coatomer epsilon subunit
*Glyma.16G155500*	AT2G28370.1	31,572,611	31,575,650	Uncharacterized protein family (UPF0497)
*Glyma.01G221600*	AT5G41460.1	55,067,816	55,073,251	Protein of unknown function (DUF604)
*Glyma.20G148400*	AT3G22640.1	38,684,625	38,687,183	Cupin family protein
*Glyma.19G170100*	AT5G43940.1	43,081,671	43,084,907	GroES-like zinc-binding dehydrogenase family protein
*Glyma.08G027600*	AT5G35360.1	2,200,021	2,207,704	Acetyl Co-enzyme a carboxylase biotin carboxylase subunit
*Glyma.11G211900*	AT1G28520.1	30,435,707	30,438,967	Vascular plant one zinc finger protein
*Glyma.08G021100*	/	1,709,026	1,709,853	/
*Glyma.08G206000*	AT1G15270.1	16,669,079	16,671,058	Translation machinery associated TMA7
*Glyma.01G230200*	AT3G03150.1	55,772,244	55,776,690	/
*Glyma.11G013200*	AT3G03150.1	916,689	922,046	/
*Glyma.18G177000*	AT4G37990.1	42,202,681	42,208,498	Elicitor-activated gene 3-2
*Glyma.01G230200*	AT3G03150.1	55,772,244	55,776,690	/

## Data Availability

Data are contained within the article and Supplementary Materials.

## Supplementary Materials

The following supporting information can be downloaded at: https://www.mdpi.com/article/10.3390/plants14233612/s1, Figure S1: GO enrichment analysis results. A: GO enrichment analysis loop; B: Top 20 pathway enrichment results; C: GO level 2. Figure S2: KEGG enrichment analysis results. Figure S3: 2% Agarose gel electrophoresis. A: 2% Agarose gel electrophoresis of Dongnong L10 root DNA; B: Promoter cloning electrophoresis strips; C: Genetic transformation single colony bacteriophage PCR validation strips. Figure S4: *GmPP2C-37like* overexpression vector and subcellular localization vector construction. A: P:pCAMBIA3300 plasmid, 1–2 linearized pCAMBIA3300 plasmid; B: P:pCAMBIA1302 plasmid, 1–2 linearized pCAMBIA1302 plasmid; C: 1–3 overexpression PCR specifically amplifying *GmPP2C-37like*, 4–7 subcellular localization PCR specifically amplifying *GmPP2C-37like*; D: 1–3 PCR results of recombinant pCAMBIA3300-*GmPP2C-37like* bacteriophage; 4–6: PCR results of recombinant pCAMBIA3300-*GmPP2C-37like* bacteriophage; M: DL2000 Marker. Figure S5: Analysis of *GmPP2C-37like* bioinformatics. A: Protein signal peptide; B: Protein hydrophilicity; C: Protein transmembrane analysis; D: Protein secondary structure; E: Phosphorylation site; F: Protein tertiary structure. Figure S6: *GmPP2C-37like* phylogenetic tree homology related to genes-Bootstrap values: Black > 90%; 90% > gray > 70%. Figure S7: pGBKT_7_-*GmPP2C-37like* vector homologous recombination. A: Physicogram of pGBKT_7_; B: Growth of transformed yeast with SD/-Trp plate; C: Linearization of two enzyme plasmids, *ECO*R I and *Bam*H I; D: PCR results of yeast Y_2_H Gold bacteriophage. Figure S8: Relative gene expression analysis. Table S1: Name and dosage of the reagents. Table S2: Partial connection system. Table S3: Name and dosage of the reagents. Table S4: Name and dosage of the reagents. Table S5: Masterbatch osmotic buffer. Table S6: Name and dosage of the reagents. Table S7: Name and dosage of the reagents. Table S8: Candidate genes to be verified. Table S9: *T*-test analysis of variance. Table S10: Primer Information. Table S11: *GmMYB29* related pathway enrichment results. Table S12: Candidate genes co-enriched by combined RNA-Seq and CHIP-Seq. Table S13: The average number of female plant (A·cm^−1^). Table S14: Paired samples *t*-test result. Table S15: Information on the distribution of gene sequences between *OE-GmMYB29* and wild-type soybean lines.
