# Supplementary material for: Transcription Factor GmMYB29 Activates GmPP2C-37like Expression to Mediate Soybean Defense Against Heterodera glycines Race 3
Source: Plants (Basel). 2025 Nov 26;14(23):3612. doi: 10.3390/plants14233612 (PMC12694451; doi:10.3390/plants14233612)
Supplement: Supplementary file 1 [file plants-14-03612-s001.zip › F2.Supplementary Picture (3).pdf]

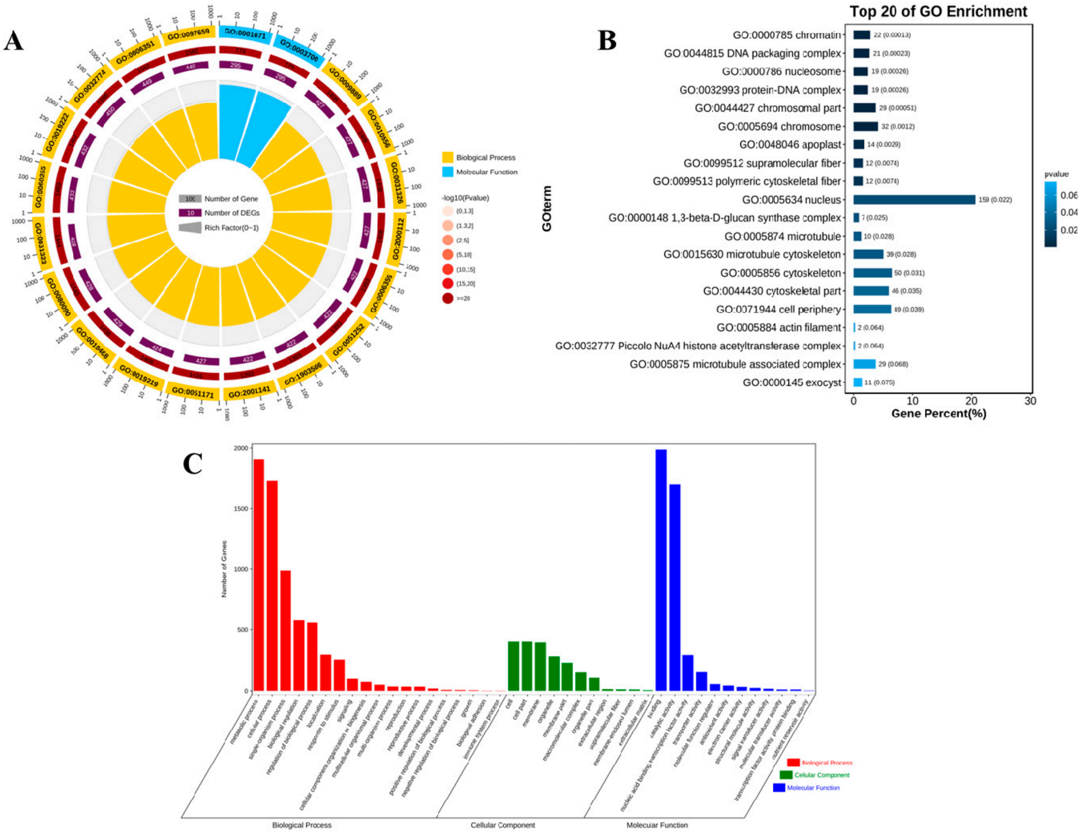

Figure S1 GO enrichment analysis results

A: GO enrichment analysis loop B: Top 20 pathway enrichment results C: GO level 2

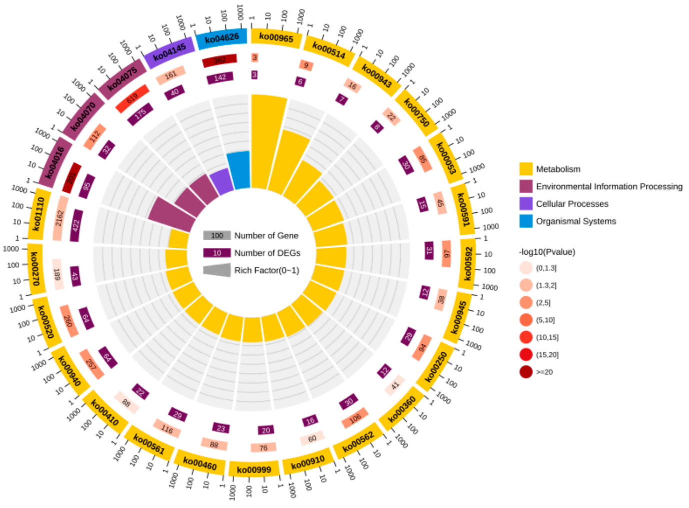

Figure S2 KEGG enrichment analysis results

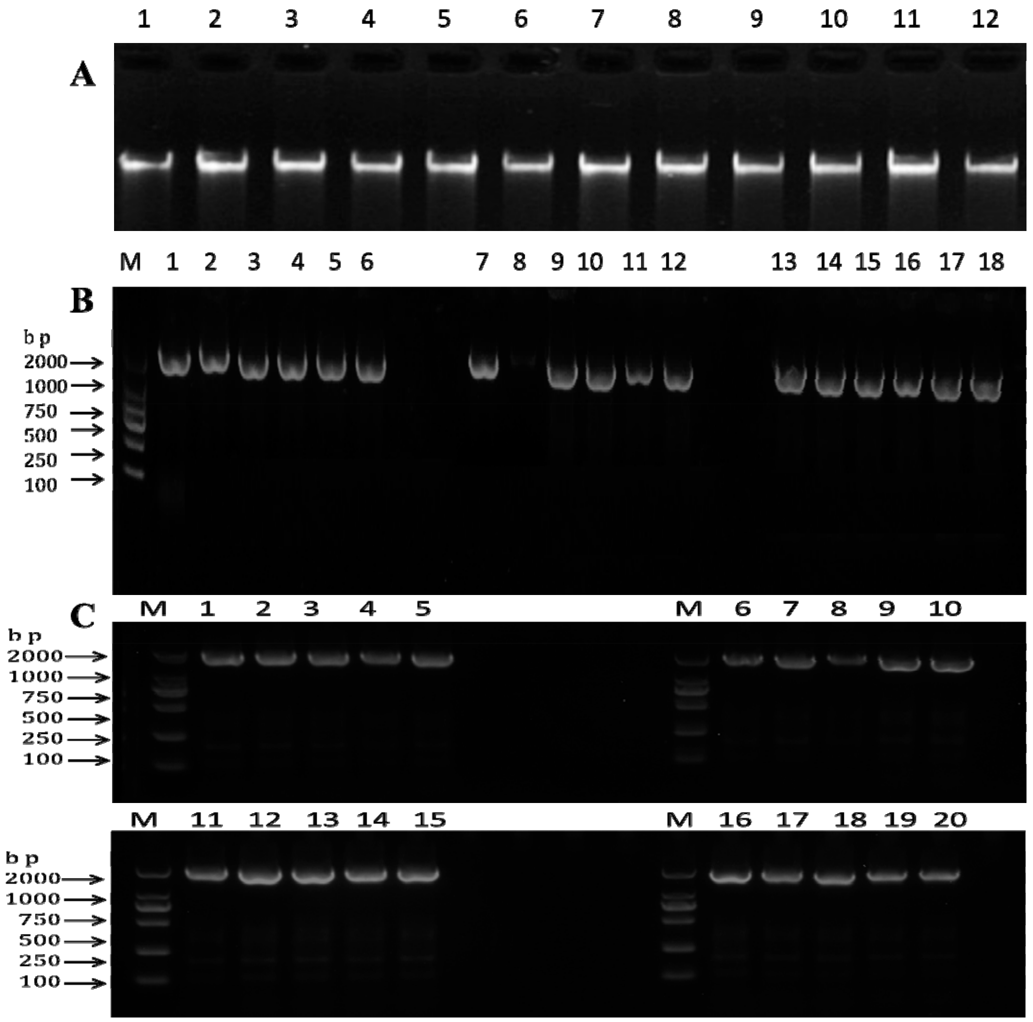

Figure S3 2 % Agarose gel electrophoresis

A：2 % Agarose gel electrophoresis of Dongnong L10 root DNA；B: Promoter cloning electrophoresis strips; C：Genetic transformation single colony bacteriophage PCR validation strips

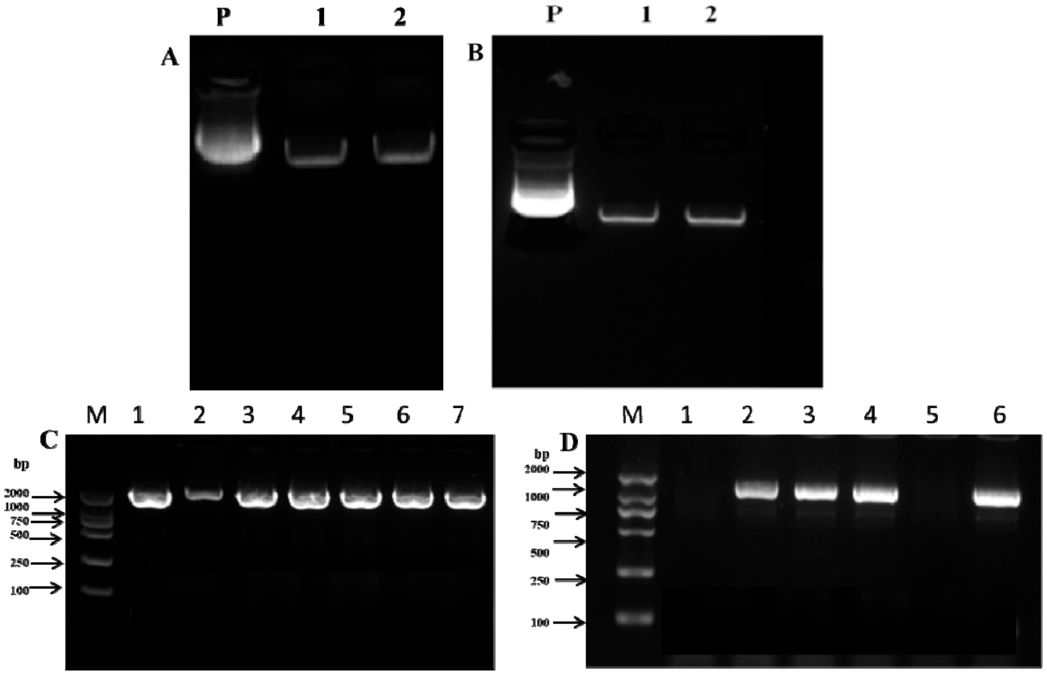

Figure S4 *GmPP2C-37like* overexpression vector and subcellular localisation vector construction

A: P:pCAMBIA3300 plasmid, 1-2 linearised pCAMBIA3300 plasmid; B : P:pCAMBIA1302 plasmid, 1-2 linearised pCAMBIA1302 plasmid; C: 1-3 overexpression PCR specifically amplifying *GmPP2C-37like*, 4-7 subcellular localisation PCR specifically amplifying *GmPP2C- 37like*; D: 1-3 PCR results of recombinant pCAMBIA3300-*GmPP2C-37like* bacteriophage; 4-6: PCR results of recombinant pCAMBIA3300-*GmPP2C-37like* bacteriophage; M: DL2000 Marker

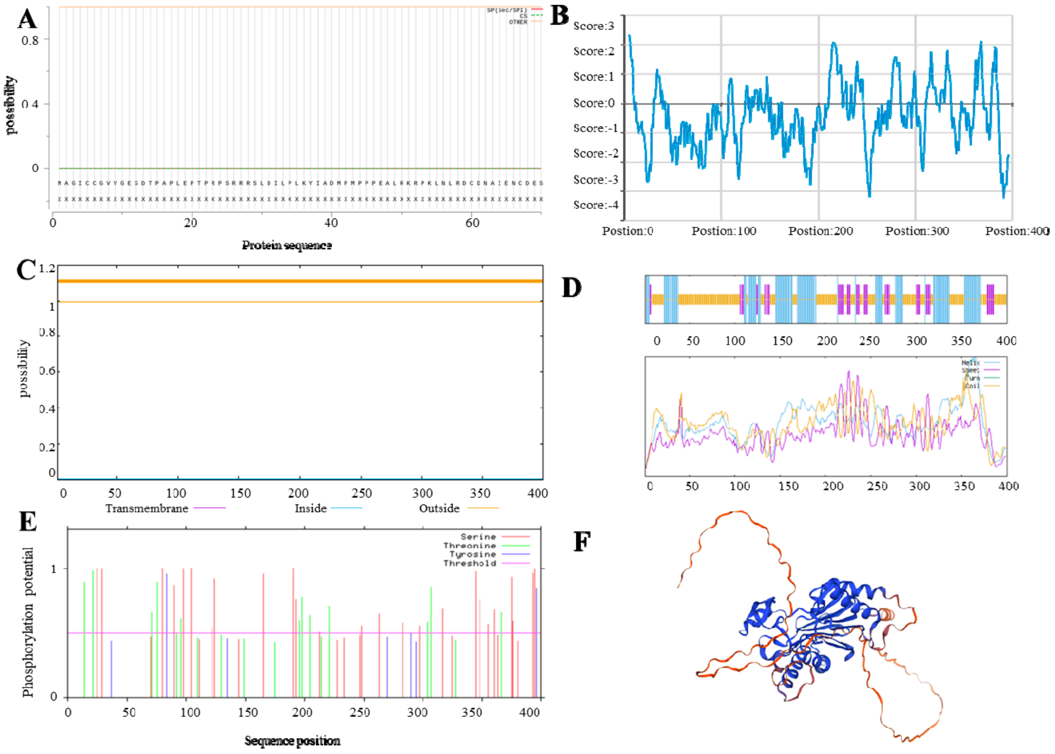

Figure S5 Analysis of *GmPP2C-37like* bioinformatics

A: protein signal peptide; B protein hydrophilicity; C: protein transmembrane analysis; D: protein secondary structure; E phosphorylation site; F: protein tertiary structure

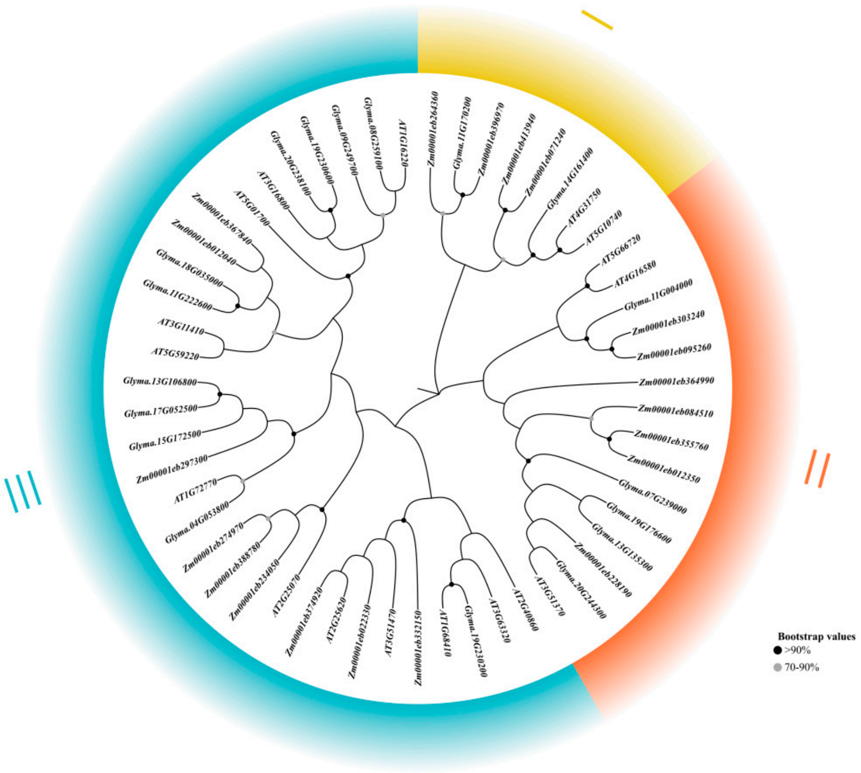

Figure S6 *GmPP2C-37like* phylogenetic tree

Homology related to genes-Bootstrap values: Black > 90%; 90%>grey>70%

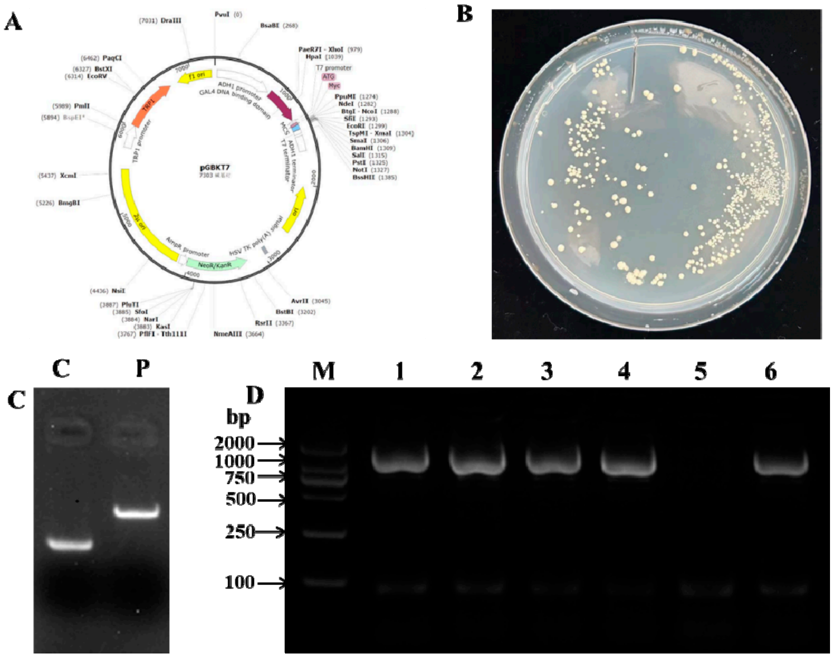

Figure S7 pGBKT$_7$-*GmPP2C-37like* vector homologous recombination

A: physicogram of pGBKT7; B: growth of transformed yeast with SD/-Trp plate; C: linearisation of two enzyme plasmids, *ECO*R I and *Bam*H I: D: PCR results of yeast Y_2H Gold bacteriophage.

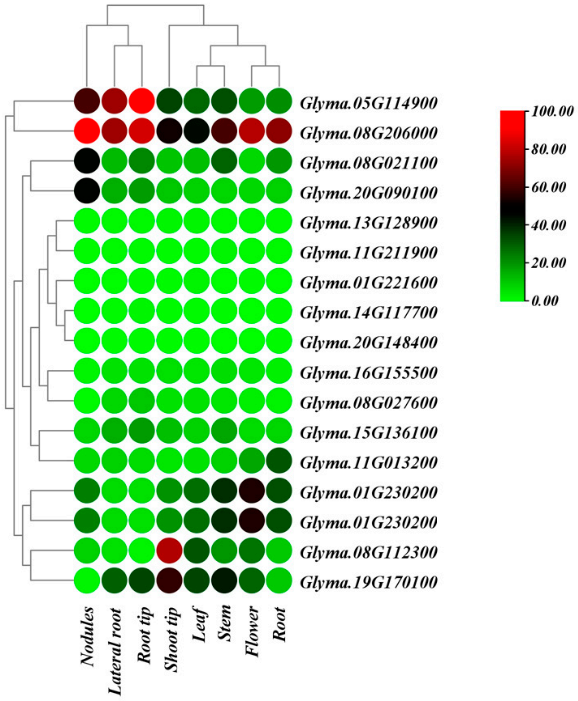

Figure S8 Relative gene expression analysis
