# Supplementary material for: Transcription Factor GmMYB29 Activates GmPP2C-37like Expression to Mediate Soybean Defense Against Heterodera glycines Race 3
Source: Plants (Basel). 2025 Nov 26;14(23):3612. doi: 10.3390/plants14233612 (PMC12694451; doi:10.3390/plants14233612)
Supplement: Supplementary file 1 [file plants-14-03612-s001.zip › F3.Supplementary Table.pdf]

Table S1 Name and dosage of the reagents

Reagents	Consumpation
Plasmid linearization	
pGreenII-0800-LUC plasmid（500ng/μL）	4 μL
Hind III Ligase	2 μL
10× buffer	4 μL
ddH_2O	10 μL
Plasmid linearization	
pCAMBIA3300 plasmid（400ng/μL）	5 μL
*Hin*d III Ligase	2 μL
10× buffer	4 μL
ddH_2O	9 μL

Table S2 Partial connection system

Reagents	Consumpation
Plasmid linearization	
promoter fragment（40 ng/μL）	2.6 μL
Linearised pGreenII 0800-LUC vector（50 ng/μL）	1.6 μL
Exnase II Ligase	2 μL
5× buffer	4 μL
ddH_2O	9.8μL
Connection system	
CDs fragment（40 ng/μL）	2.6 μL
Linearised pCAMBIA3300 vector（50 ng/μL）	1.6 μL
Exnase II Ligase	2 μL
5× buffer	4 μL
ddH_2O	9.8μL

Table S3 Name and dosage of the reagents

Reagents	Consumpation
Plasmid linearization	
pAbAi plasmid（385.6ng/μL）	7.5 μL
*Xho*ILigase	3 μL
*Sma*ILigase	6 μL
10× buffer	10 μL
ddH_2O	73.5 μL
Connection system	
promoter fragment（185 ng/μL）	0.45 μL
Linearised pAbAi vector（37 ng/μL）	2.65 μL
Exnase II Ligase	2 μL
5× buffer	4 μL

ddH_2O	9.8μL

Table S4 Name and dosage of the reagents

Reagents	Consumpation
Plasmid linearization	
pGAD-T_7 plasmid（406ng/μL）	6.2 μL
ECOR I Ligase	3 μL
*Bam*HI Ligase	6 μL
10× buffer	10 μL
ddH_2O	74.8 μL
Connection system	
promoter fragment（27.5 ng/μL）	1.2 μL
Linearised pGAD-T_7 vector（50 ng/μL）	3.2 μL
Exnase II Ligase	2 μL
5× buffer	4 μL
ddH_2O	9.6μL

Table S5 Masterbatch osmotic buffer

Reagents	Content
500 mM MES	1mL
1 mM $MgCl_2$	1mL
7.5μL Acetosyringone(As)	7.5μL
ddH_2O	Add to 50mL

Table S6 Name and dosage of the reagents

Reagents	Consumpation
Plasmid linearization	
pCAMBIA1302 plasmid（400ng/μL）	5 μL
NCO I Ligase	2 μL
10× buffer	4 μL
ddH_2O	9 μL
Connection system	
DNA fragment（40 ng/μL）	2.6 μL
Linearised pCAMBIA1302 vector（50 ng/μL）	1.6 μL
Exnase II Ligase	2 μL
5× buffer	4 μL
ddH_2O	9.8μL

Table S7 Name and dosage of the reagents

Reagents	Consumpation
Plasmid linearization	

pGAD-T_7 plasmid（417ng/μL）	6 μL
*ECOR*ILigase	3 μL
*BamH*ILigase	6 μL
10× buffer	10 μL
ddH_2O	25μL
Connection system	
DNA fragment（50 ng/μL）	1.2μL
Linearised pGBKT$_7$ vector（67 ng/μL）	2.2 μL
Exnase II Ligase	2 μL
5× buffer	4 μL
ddH_2O	10.6μL

Table S8 Candidate genes to be verified

Chr.	Distance to TSS location	Gene ID	Gene annotation	Arabidopsis ID	Enrichment pathways
Chr01	-283	*Glyma.01G222300*	WRKY transcription factor 22	*AT4G01250*	Plant-pathogen interaction
Chr18	0	*Glyma.18G087800*	RPM1, RPS3; disease resistance protein RPM1		Plant-pathogen interaction
Chr06	0	*Glyma.06G187200*	EDS1; enhanced disease susceptibility 1 protein	*AT3G48080*	Plant-pathogen interaction
Chr07	-93	*Glyma.07G202300*	cytochrome P450, family 93, subfamily D, polypeptide 1	*AT5G06900*	Isoflavonoid biosynthesis
Chr03	298	*Glyma.18G267900*	isoflavone-7-O-methyltransferase	*AT4G35150*	Isoflavonoid biosynthesis
Chr18	0	*Glyma.18G035000*	PP2C	*AT3G11410*	MAPK signaling pathway- plant
Chr08	630	*Glyma.08G223400*	MKK4_5	*AT1G51660*	MAPK signaling pathway- plant
Chr15	-1909	*Glyma.15G051600*	BAK1	*AT4G33430*	MAPK signaling pathway- plant
Chr15	-1479	*Glyma.15G062400*	PR1	*AT2G14580*	Plant hormone signal transduction
Chr02	-820	*Glyma.02G125600*	GH3	*AT2G14960*	Plant hormone signal transduction
Chr05	-1473	*Glyma.05G081900*	SNRK2	*AT5G66880*	Plant hormone signal transduction
Chr13	-98	*Glyma.13G030300*	LOX2s	*AT3G45140*	Plant hormone signal transduction

Table S9 T-test analysis of variance

Treatments	average value	standard deviation	t-value	Mean P-value (two-tailed)
GmPP2C-37like -OX	2.560	0.651	4.844	0.000
WT	4.127	1.070		

Note: *GmPP2C-37like-ox*: transgenic soybean overexpressing the *GmPP2C-37like* gene; WT: Wild-type plants

Table S10 Primer Information

Primer name	Gene ID	5'-3' Upstream primer	5'-3' Downstream primer	Fuction
Luc-1	*Glyma.01G222300*	gtcgacggtatcgat*aagctt*AAGGCCACTCGAAACCAATT	caggaattcgatatc*aagctt*CAACTCGCATGGAGGGTTTT	LUC
Luc-2	*Glyma.18G087800*	gtcgacggtatcgat*aagctt*ACAAACTTATTATTATTAAT	caggaattcgatatc*aagctt*GGTTTGTTGTGATTGCAAAA	LUC
Luc-3	*Glyma.06G187200*	gtcgacggtatcgat*aagctt*ACTTCATATTTTAAACAAAA	caggaattcgatatc*aagctt*CATGTGTATAGTTGTGTGAA	LUC
Luc-4	*Glyma.07G202300*	gtcgacggtatcgat*aagctt*TCACTGGCTGTCTGTCTTGA	caggaattcgatatc*aagctt*GGTCTACCTACACTTATCAA	LUC
Luc-5	*Glyma.18G267900*	gtcgacggtatcgat*aagctt*ACGCCAACATAGCCAATCGA	caggaattcgatatc*aagctt*AATTGCAAACGAAGTTTGAT	LUC
Luc-6	*Glyma.18G035000*	gtcgacggtatcgat*aagctt*TTCTTTGATGATTTGATGAG	caggaattcgatatc*aagctt*TGAGATTGTAAATTGTGCTA	LUC
Luc-7	*Glyma.08G223400*	gtcgacggtatcgat*aagctt*CTAAAAATATAGATAGTGAA	caggaattcgatatc*aagctt*AGGAAGGGAGAGGAGGGTGG	LUC
Luc-8	*Glyma.15G051600*	gtcgacggtatcgat*aagctt*TATATGAATGACTTGGTCAG	caggaattcgatatc*aagctt*CTTTATGAATTATGAGGTAT	LUC
Luc-9	*Glyma.15G062400*	caggaattcgatatc*aagctt*TGATGTATTTTATTTAGATA	caggaattcgatatc*aagctt*AAGGTTGGAGCTCCTTTGAG	LUC
Luc-10	*Glyma.02G125600*	caggaattcgatatc*aagctt*TTATCTCACGTGCAAATAAA	caggaattcgatatc*aagctt*AGAGTTTATATAGAGGAAGA	LUC
Luc-11	*Glyma.05G081900*	caggaattcgatatc*aagctt*ACAAAACTGAGAGCACCTCA	caggaattcgatatc*aagctt*GATATTTTTATCACCACCAC	LUC
Luc-12	*Glyma.13G030300*	caggaattcgatatc*aagctt*GGGGTAGGATTGCATCATCG	caggaattcgatatc*aagctt*TATTGTAAAGTTTGAGAACT	LUC
pGADTMYB29	*pGADTMYB29*	GCCATGGAGGCCAGT*GAATTCA*TGGTGAGAGCTCCTTGTTG	CAGCTCGAGCTCGAT*GGATCC*TCAGAACTCTGACAATTCTA	yeasts
Abai-Gmpp2c	*Abai-Gmpp2c*	aattcgagctcggta*cccggg*TTCTTTGATGATTTGATGAG	atacagagcacatgc*ctcgag*TGAGATTGTAAATTGTGCTA	yeasts
OE-PP2CC	*OE-PP2CC*	TCGAGCTCCGTCGAC*AAGCTT*ATGGCTGGAATTTGCTGTGG	GCCCTTGCTCACCAT*AAGCTT*ATTAACGTCGTTGTAGTTTG	OX
OE-MYB29	*OE-MYB29*	GCCATGGAGGCCAGTGAATTCATGGGAATGGTCTATGCTGAC	CGAGGTCGACGGATCCGCTTTCAATCATTGGTTCGGG	OX
GmMYB29-RNAi-1	*GmMYB29*-RNAi-1	TTACATTTACAATTACCATGGATGGTGAGAGCTCCTTGTTG	TCGATTGGGCGCGCCCCATGGGGCTTAGTGGCTCTTTTGGA	RNAi
GmMYB29-RNAi-2	*GmMYB29-RNAi-2*	TGGATCCTAGGTGAGTCTAGAGGCTTAGTGGCTCTTTTGG	GGTCTTAATTAACTCTCTAGAATGGTGAGAGCTCCTTGTTG	RNAi

		A		
Sub-PP2CC	*sub-PP2CC*	ACGGGGGACTCTTGA*ccatgg*ATGGCTGGAATTTGCTGTGG	tactagtcagatcta*ccatgg*ATTAACGTCGTTGTAGTTTG	Sub
qPCR-PP2C	qPCR-PP2C	CGTCGATTCTGTTGACCAAGT	GTCGTTGATCCCTCCTCAAATC	qPCR
Actin 4	*Actin 4*	GTTTCAAGCTCTTGCTCGTAATCA	GTGTCAGCCATACTGTCCCCATTT	qPCR
BGKBD-PP2C	*BGKBD-PP2C*	CATGGAGGCC*GAATTC*ATGGCTGGAATTTGCTGTGG	GCAGGTCGAC*GGATCC*TTAATTAACGTCGTTGTAGT	yeasts
Bar	*Bar*	TCAAATCTCGGTGACGGGC	ATGAGCCCAGAACGACGC	LUC
LUC-Ⅰ	*LUC*-Ⅰ	gtcgacggtatcgataagcttTTATCAATAAGGTACTTTACAGATA	caggaattcgatatcaagcttGAGATTGTAAATTGTGCTAGAGAAG	
LUC-Ⅱ	*LUC*-Ⅱ	gtcgacggtatcgataagcttTCCAAATAGCCCAGAT	caggaattcgatatcaagcttGAGATTGTAAATTGTGCTAGAGAAG	LUC
LUC-Ⅲ	*LUC*-Ⅲ	gtcgacggtatcgataagcttATAAAGCAAGTGTCGC	caggaattcgatatcaagcttGAGAAGGAAAGAGGAA	LUC
LUC-Ⅳ	*LUC*-Ⅳ	gtcgacggtatcgataagcttAGGGAAAGCAAATGAG	caggaattcgatatcaagcttGAGATTGTAAATTGTGCTAGAGAAG	LUC

Note: Underlined are homology arm sequences; italics are enzyme cleavage site sequences; double underlined and bolded are clone sequences

Table S11 *GmMYB29* related pathway enrichment results

Pathway	Candidate genes with pathway annotation (1480)	All genes with pathway annotation (8179)	Pvalue	Qvalue	Pathway ID
Plant-pathogen interaction	142 (9.59%)	362 (4.43%)	0.000000	0.000000	ko04626
MAPK signaling pathway - plant	95 (6.42%)	201 (2.46%)	0.000000	0.000000	ko04016
Plant hormone signal transduction	175 (11.82%)	619 (7.57%)	0.000000	0.000000	ko04075
Ascorbate and aldarate metabolism	30 (2.03%)	85 (1.04%)	0.000113	0.003688	ko00053
alpha-Linolenic acid metabolism	31 (2.09%)	97 (1.19%)	0.000653	0.017114	ko00592
Other types of O-glycan biosynthesis	6 (0.41%)	9 (0.11%)	0.001771	0.033325	ko00514
Alanine, aspartate and glutamate metabolism	29 (1.96%)	94 (1.15%)	0.001781	0.033325	ko00250
Phenylpropanoid biosynthesis	64 (4.32%)	257 (3.14%)	0.003435	0.056253	ko00940
Phosphatidylinositol signaling system	32 (2.16%)	112 (1.37%)	0.004130	0.059902	ko04070

Amino sugar and nucleotide sugar metabolism	64 (4.32%)	260 (3.18%)	0.004573	0.059902	ko00520
Betalain biosynthesis	3 (0.2%)	3 (0.04%)	0.005915	0.068084	ko00965
Inositol phosphate metabolism	30 (2.03%)	106 (1.3%)	0.006237	0.068084	ko00562
Linoleic acid metabolism	15 (1.01%)	45 (0.55%)	0.010210	0.102888	ko00591
Isoflavonoid biosynthesis	7 (0.47%)	16 (0.2%)	0.015648	0.146422	ko00943
Phagosome	40 (2.7%)	161 (1.97%)	0.018793	0.164122	ko04145
Biosynthesis of secondary metabolites	422 (28.51%)	2162 (26.43%)	0.024797	0.203027	ko01110
Stilbenoid, diarylheptanoid and gingerol biosynthesis	12 (0.81%)	38 (0.46%)	0.031374	0.239327	ko00945
Vitamin B6 metabolism	8 (0.54%)	22 (0.27%)	0.032885	0.239327	ko00750
Cyanoamino acid metabolism	23 (1.55%)	88 (1.08%)	0.037917	0.248363	ko00460
Glycerolipid metabolism	29 (1.96%)	116 (1.42%)	0.037918	0.248363	ko00561
Biosynthesis of various plant secondary metabolites	20 (1.35%)	76 (0.93%)	0.047340	0.295312	ko00999
Phenylalanine metabolism	12 (0.81%)	41 (0.5%)	0.054456	0.324262	ko00360
Cysteine and methionine metabolism	43 (2.91%)	189 (2.31%)	0.059255	0.325225	ko00270
Nitrogen metabolism	16 (1.08%)	60 (0.73%)	0.063934	0.325225	ko00910
beta-Alanine metabolism	22 (1.49%)	88 (1.08%)	0.064391	0.325225	ko00410

Table S12 Candidate genes co-enriched by combined RNA-Seq and ChIP-Seq

ID	Description	pvalue	geneID	Count
ko00910	Nitrogen metabolism	5.11E-07	*Glyma.02G213700/Glyma.06G109200/Glyma.06G182700/Glyma.08G286700/Glyma.11G195200/Glyma.13G084000/Glyma.14G165000/Glyma.16G153400*	8
ko00250	Alanine, aspartate and glutamate metabolism	1.86E-05	*Glyma.02G228100/Glyma.04G007300/Glyma.04G042100/Glyma.11G170300/Glyma.11G171400/Glyma.16G153400/Glyma.18G061100/Glyma.18G116900*	8
ko00943	Isoflavonoid biosynthesis	0.000396	*Glyma.01G172600/Glyma.03G147700/Glyma.09G049100/Glyma.10G176700/Glyma.20G213700*	5
ko00940	Phenylpropanoid biosynthesis	0.005106	*Glyma.01G130800/Glyma.01G187700/Glyma.03G038500/Glyma.03G038700/Glyma.05G147000/Glyma.09G022300/Glyma.09G277800/Glyma.15G128700/Glyma.16G055900*	9
ko00960	Tropane, piperidine and pyridine alkaloid biosynthesis	0.005348	*Glyma.06G235500/Glyma.12G059100/Glyma.12G059200/Glyma.19G105100*	4
ko00130	Ubiquinone and other terpenoid-quinone biosynthesis	0.00582	*Glyma.01G134600/Glyma.06G235500/Glyma.10G070200/Glyma.11G210400/Glyma.20G245100*	5
ko04016	MAPK signaling pathway - plant	0.012747	*Glyma.02G042500/Glyma.10G007000/Glyma.13G251600/Glyma.13G252400/Glyma.15G062400/Glyma.15G062500/Glyma.16G084700/**Glyma.18G035000***	8
ko00052	Galactose metabolism	0.024328	*Glyma.06G191500/Glyma.13G349300/Glyma.15G024600/Glyma.17G138500*	4
ko00941	Flavonoid biosynthesis	0.03602	*Glyma.01G187700/Glyma.05G022100/Glyma.05G147000/Glyma.19G105100*	4

Table S13 The average number of female plant (A·cm- 1)

Treatments	P1	P2	P3	P4	P5	P6	P7	P8	P9	P10	P11	P12	P13	P14	P15
GmMYB29-RNAi	2	3.1	3.0	2.3	2.1	2.4	4	4.1	3.2	4.3	5.33	3.2	5.5	6.4	5.6
GmMYB29-pCAMBIA3300	1.7	2.7	1.8	1.9	2.9	1.8	2.0	2.2	2.4	3	3.5	2.7	3.6	2.7	2.9
pCAMBIA3300	4.7	1.1	1.8	2.1	2.4	1.7	3.3	1.1	2.5	2.44	1.9	4.0	3.8	2.2	3.4

P is for plant; GmMYB29-pCAMBIA3300: Transgenic soybean overexpressing *GmMYB29* gene. GmMYB29-RNAi: RNAi interferes with *GmMYB29* expression in transgenic soybean. pCAMBIA3300: Control soybean transferred into empty carrier.

Table S14 Paired samples t test result

Treatments	Average	Variance	Observed value	Poisson correlation coefficient	P (T < = t) single tail	T single tail criticality	P 0.01（sig）
GmMYB29-RNAi	3.79	1.07	15	0.14	3.31E-03	2.14	0.007
GmMYB29-pCAMBIA3300	1.83	0.64	15	-0.33	4.61E-03	2.14	0.004
pCAMBIA3300	2.60	1.36	15	-	-	-	-

GmMYB29-pCAMBIA3300: Transgenic soybean overexpressing *GmMYB29* gene. *GmMYB29*-RNAi: RNAi interferes with *GmMYB29* expression in transgenic soybean. pCAMBIA3300: Control soybean transferred into empty carrier.

Table S15 Information on the distribution of gene sequences between OE-*GmMYB29* and wild-type soybean lines

Sample name	Number of gene enrichments	0-1 kb promoter region(%)	1-2 kb promoter region(%)	2-3 kb promoter region(%)	5' UTR(%)	3' UTR(%)	Distal intergenic region(%)	0-2 kb promoter region(%)
WT	63936	40.87%	12.32%	6.98%	0.06%	0.49%	37.60%	53.19%
OE-GmMYB29	3279	23.58%	6.78%	4.64%	0.06%	0.31%	62.87%	30.36%
